# Nanometer-resolution tracking of single cargo reveals dynein motor mechanisms

**DOI:** 10.1038/s41589-024-01694-2

**Published:** 2024-08-01

**Authors:** Chunte Sam Peng, Yunxiang Zhang, Qian Liu, G. Edward Marti, Yu-Wen Alvin Huang, Thomas C. Südhof, Bianxiao Cui, Steven Chu

**Affiliations:** 1 Department of Physics, Stanford University, Stanford, CA USA; 2 Department of Molecular and Cellular Physiology, Stanford University, Stanford, CA USA; 3 Howard Hughes Medical Institute, Stanford University, Stanford, CA USA; 4 Department of Chemistry, Stanford University, Stanford, CA USA; 5 Present Address: Department of Chemistry, Massachusetts Institute of Technology, Cambridge, MA USA; 6 Present Address: Broad Institute of MIT and Harvard, Cambridge, MA USA; 7 Present Address: Department of Chemistry and Shanghai Key Laboratory of Molecular Catalysis and Innovative Materials, Fudan University, Shanghai, China; 8 Present Address: Department of Molecular Biology, Cell Biology, and Biochemistry, Brown University, Providence, RI USA

**Keywords:** Single-molecule biophysics, Chemical biology

## Abstract

Cytoplasmic dynein is essential for intracellular transport. Despite extensive in vitro characterizations, how the dynein motors transport vesicles by processive steps in live cells remains unclear. To dissect the molecular mechanisms of dynein, we develop optical probes that enable long-term single-particle tracking in live cells with high spatiotemporal resolution. We find that the number of active dynein motors transporting cargo switches stochastically between one and five dynein motors during long-range transport in neuronal axons. Our very bright optical probes allow the observation of individual molecular steps. Strikingly, these measurements reveal that the dwell times between steps are controlled by two temperature-dependent rate constants in which two ATP molecules are hydrolyzed sequentially during each dynein step. Thus, our observations uncover a previously unknown chemomechanical cycle of dynein-mediated cargo transport in living cells.

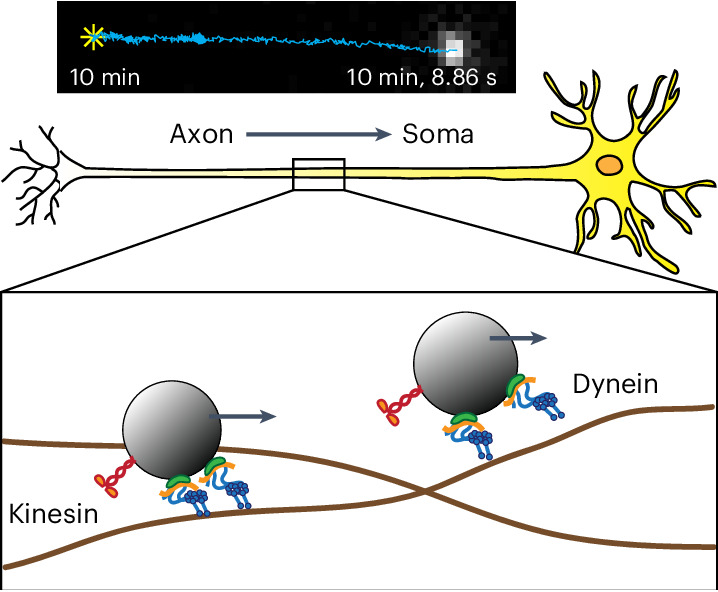

## Abstract (web summary)

How dynein moves in living cells remains unclear. Here the authors develop a photostable probe that enables long-term single-cargo tracking in living neurons and find the number of active dynein motors per cargo switches between one and five. Moreover, two ATPs are hydrolyzed during each dynein step.

## Main

**Fig. 1:**
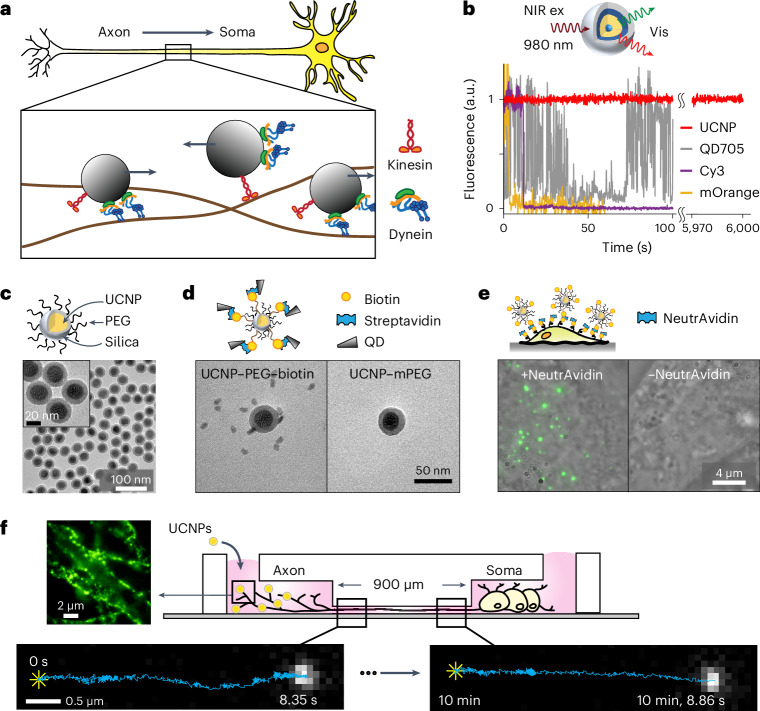
UCNPs used in single-particle imaging. **a**, Schematic representation of axonal transport, which involves endosomes being transported by kinesin and dynein motors. **b**, Top, schematic design of the silica-coated CSS UCNPs used in live-neuron SPT. Bottom, fluorescence time traces of single UCNP (red), QD705 (gray), Cy3 dye (purple) and mOrange fluorescent protein (orange). **c**, TEM images of the PEGylated NaYF_4_:20% Yb^3+^, 2% Er^3+^@SiO_2_ (*n* = 10 independent experiments). **d**, Satellite assembly of UCNPs and QDs via biotin–streptavidin binding. TEM images of a mixture of streptavidin-conjugated QD705 and UCNPs bearing biotin (left) and without biotin (right; *n* = 3 independent experiments). **e**, Live-cell imaging of btn-UCNPs on HeLa cells whose membrane proteins have been biotinylated. Overlay of brightfield images of HeLa cells and maximum-intensity-projected luminescence images of btn-UCNPs in the presence (left) and absence (right) of NeutrAvidin (*n* = 20 cells). **f**, Schematic design of single-cargo tracking during axonal transport in live neurons. Neurons are cultured in microfluidic devices with 900-μm-long microchannels. Each field of view observes transport over 5–15 μm. WGA-conjugated CSS UCNPs are incubated in the axon chamber to induce endocytosis. Representative luminescence image of DRG neuron axons labeled with UCNPs (left). Bottom, representative luminescence images of the same cargo during axonal transport from the distal axon (*t* = 0 s, left) to near-soma (*t* ≈ 10 min, right). Single-particle trajectories are overlaid on the fluorescent images where the star (✳) indicates the start positions. btn, biotinylated.

Dynein molecular motors have key roles in essential cellular processes, such as cargo transport and cilia motility. Cytoplasmic dynein is a microtubule-based motor used in many eukaryotic cells for most of the retrograde transport toward the central portion of the cell (Fig. 1a). On the other hand, proteins synthesized in the cell body are anterogradely transported to the cell periphery by kinesin motors.

Dynein, discovered 35 years ago^1^, belongs to the family of AAA+ ATPases (for ATPases associated with diverse activities), which typically unfold proteins and dismantle macromolecular complexes and aggregates. This class of molecular motors, which include N-ethylmaleimide-sensitive factor and ClpXP protease, transduce chemical energy from the hydrolysis of multiple ATP molecules into mechanical work although the ATP hydrolysis can be sequential or stochastic^2^. The dynein motor consists of two identical heavy chains and several intermediate, light intermediate and light chains, with a total mass of ~1.4 MDa. Each heavy chain contains a ring of six AAA+ modules and a set of allosteric interacting appendages^3^. The dynein dimer works in an assembly of light chains and associated proteins, such as dynactin adapters and Lis1 (ref. ^4^). Because of their complexity, the molecular functions of dynein-mediated transport are incompletely understood, and the many elegant structural and single-molecule studies of dynein over the past decades have not fully elucidated their precise biophysical mechanism of action^3,5–10^.

To maintain robust transport in cells, both dynein and kinesin motors are known to work collectively to enhance their processivity. This is especially critical for the axonal transport of biomolecules and organelles in neurons because the length of the narrow axons prohibits efficient transport by diffusion. Dysregulation of axonal transport is thought to lead to neurodegenerative disorders^11^, and robust transport relies ultimately on the fundamental biophysical properties of these motors. While it is recognized that multiple dyneins are associated with cellular vesicles, it is not known how many dynein motors are actively transporting the cargos in live cells or if the number of active motors changes during the retrograde transport. Moreover, most of our knowledge of the molecular kinetics of dynein motility has been deduced from in vitro single-molecule studies^12^ where individual steps were resolved by lowering the ATP concentration to the few μm range. At these low concentrations, the binding time of ATP is the rate-limiting step. In contrast, cellular ATP concentrations are typically three orders of magnitude higher than used in the in vitro studies, leading to cargo transport speeds faster than 1 μm s^−1^ (refs. ^2–14^), which corresponds to stepping rates of more than 100 steps per second. As a result, the crucial molecular dynamics under cellular conditions with high ATP concentrations might have been overlooked.

Single-molecule fluorescence microscopy has been an essential tool in capturing the structure, interactions and dynamics of molecular machines^12,13^. However, many currently used fluorescent probes are not ideal. Organic fluorophores or fluorescent proteins exhibit fluorescence intermittency (blinking) and irreversible photobleaching within tens of seconds (Fig. 1b). Numerous strategies have been developed to reduce fluorophore photobleaching and blinking, such as using enzymatic oxygen-scavenging systems^14^, adding triplet-state quenchers^14,15^, using low concentrations (2%) of dissolved oxygen^16^ or using a scaffold that recruits multiple fluorescent proteins or dyes^17–19^. However, these methods are still limited to the timescale of a few minutes. Quantum dots (QDs) are more photostable, but their blinking prevents continuous imaging (Fig. 1b). While blinking has been mitigated in recent work, it has not been fully eliminated^20,21^.

In this work, we describe how our development and application of substantially improved, fully photostable optical probes allow us to track the dynein transport of 12 individual cargos over 900 μm for tens of minutes. This capability enables us to deduce that the number of actively engaged motors changed during the journey. While some vesicles use two motors, other cargos are found to simultaneously engage as many as five motors. Our bright optical probes also enable the resolution of individual molecular steps in live cells. The ability to measure dwell time between molecular steps in live cells reveals that dynein stepping is accurately described by two equal and temperature-dependent rate constants. We show that the only rate constant consistent with this observation is the thermal desorption of the phosphate. Thus, our live-cell single-step measurements lead us to conclude that each dynein step requires the sequential hydrolysis of two ATPs at different AAA+ sites.

## Results

### Development of photostable and biocompatible probes

To perform long-term single-particle tracking (SPT) in live neurons, we used photostable rare-earth ion-doped upconverting nanoparticles (UCNPs). The UCNPs luminescence is extremely stable, enabling single-particle imaging for many hours (Fig. 1b and Supplementary Fig. 1) and long-term SPT in live cells^22–24^. The near-infrared (NIR) excitation into visible and UV emissions via multiphoton energy transfer among the sensitizer Yb^3+^ and emitters such as Er^3+^ and Tm^3+^ ions eliminate cellular autofluorescence, resulting in essentially background-free imaging^22,24–29^. The sharp emissions can be tuned by varying both the type and the concentration of emitter ions to perform multicolor imaging^30–32^. Moreover, the use of NIR excitation of UCNPs minimizes photoreactive toxicity^33^ and permits deep-tissue imaging^34^.

Ideally, nanoparticle functionalization should allow easy derivatization of various targeting molecules on the nanoparticle surface while maintaining low nonspecific binding to cells^35^. To this end, we used the reverse microemulsion method^36^ to grow a 5-nm thin layer of silicon dioxide on 23 nm β-NaYF_4_ nanoparticles codoped with 20% Yb^3+^ and 2% Er^3+^, resulting in monodispersed UCNPs in aqueous solution (Fig. 1c and Supplementary Figs. 2 and 3). The uniform silica coating allows direct conjugation of silane derivatives, such as silane-polyethylene glycol (PEG_500_), for mitigating nonspecific binding, and silane–PEG_3400_–biotin as the targeting group. Silica-coated UCNPs have been shown to exhibit no observable cytotoxicity^37^.

To validate the presence of the biotin moiety on the surface of the UCNPs, transmission electron microscopy (TEM) images were taken for a mixture of biotinylated UCNPs (btn-UCNPs) and streptavidin-coated QDs (Fig. 1d and Supplementary Fig. 4). The btn-UCNPs were found to be decorated with QDs, forming a satellite assembly. In contrast, PEGylated UCNPs lacking biotin ligands did not bind QDs. An in vitro single-particle binding assay on PEGylated glass coverslip was used to quantitate the degree of nonspecific sticking of PEGylated UCNPs (Supplementary Fig. 5). Specific binding of individual UCNPs via the biotin–NeutrAvidin interaction was evident, while control experiments without NeutrAvidin showed no binding. Fluorescence images of multiple fields of view established a lower boundary of 2,000:1 specific versus nonspecific binding. The specificity of btn-UCNPs was also validated on live cells (Fig. 1e and Supplementary Fig. 6).

### Single-cargo tracking over millimeter distances

The photostable and background-free UCNPs are ideal for long-term monitoring of axonal transport in live neurons at high spatiotemporal resolution. To reduce the excitation power density for live-cell imaging, we used our previously reported Yb^3+^-rich core–shell–shell (CSS) UCNPs, NaYF_4_@NaYbF_4_:8% Er^3+^@NaYF_4_ (Supplementary Fig. 7)^22^. These 28 nm diameter particles are 150× brighter at 8 W cm^−^^2^ and 6× brighter at 10 kW cm^−^^2^ than the canonical core-only NaYF_4_: 20% Yb^3+^ and 2% Er^3+^ due to the increased 980-nm light absorption by higher levels of Yb^3+^ ions, improved crystal quality and the protection against energy loss to the solvent by an inert NaYF_4_ shell.

For long-distance tracking of neuronal transport, dorsal root ganglion (DRG) neurons were cultured in compartmentalized microfluidic devices so that their axons grew aligned within microchannels^38,39^. This geometry allowed simple differentiation between retrograde and anterograde transport, which are defined as moving from the distal axon to the soma and from the soma to the distal axon, respectively (Fig. 1f). The btn-UCNPs were conjugated to wheat germ agglutinin (WGA), which targets glycosylated membrane proteins and triggers receptor-mediated endocytosis^39^. UCNP-containing endosomes that were transported by dynein motors toward the soma were imaged in the microchannels where there were no free UCNPs to interfere with the measurements. The same cargo was tracked through the ~900 μm length of the microchannel by recording several movies taken at 100 frames per second over a period of 10–15 min. Figure 1f displays the unprocessed raw images, where ~1,000 photons were collected in each video frame. Because there was no cell autofluorescence, the spatial localization resolution is expected to be shot-noise limited and estimated to be 6.4 nm (‘Live-neuron imaging’).

**Fig. 2:**
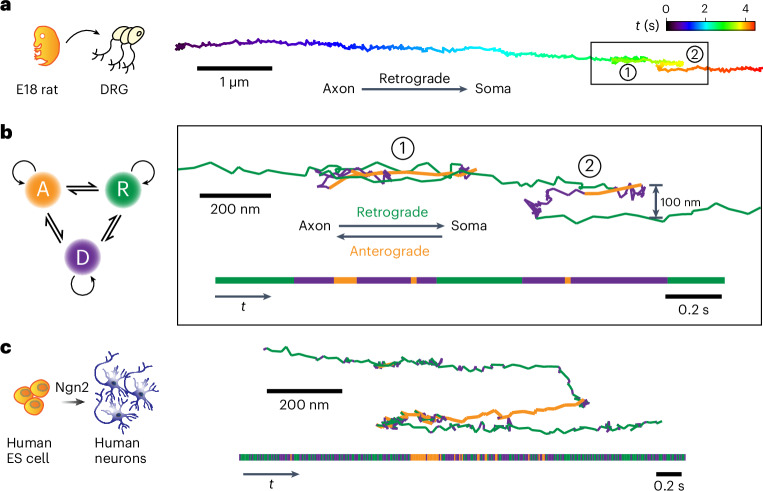
Long-term tracking of retrograde transport at millisecond and nanometer spatiotemporal resolution. **a**, An example trajectory of retrograde cargo in rat DRG neurons that is color-coded in time, showing cargo reversal and track switching (black box). **b**, HMM–Bayes analysis of the single-cargo trajectory in the boxed region in **a**. The inferred motions include one diffusive state (purple) and the following two active transport states: retrograde (green) and anterograde (orange) transport. The temporal sequence of motion states is illustrated in the horizontal bar at the bottom. The trajectory color-coded by motion states clearly shows reversal (region 1) and track switching (region 2). **c**, One example of single-cargo tracking in human-induced neurons (iNs) also illustrates track switching and rapid conversions between the diffusive, retrograde and anterograde transport states. Equal scales are used for horizontal and vertical dimensions.

The representative trajectory (Fig. 2a) reveals the dynamic and complex behavior of a single-cargo in a ‘stop-and-go’ fashion, as previously reported^38,39^. The nonblinking, photostable luminescence provides continuous records of retrograde transport that include frequent motion reversals as well as cargo switching to neighboring microtubules (Fig. 2b). To quantitatively characterize these motions, a hidden Markov modeling (HMM)–Bayes approach was used to automatically annotate heterogeneous motion states locally along a single trajectory^40^. In contrast to the commonly used mean-square displacement analysis, the HMM–Bayes method reveals stochastic switching between different motion states without the need to time-average over a substantial number of particle displacement steps. Long-term SPT is ideally suited for HMM–Bayes analysis because the accuracy of the model prediction increases with the length of the trajectory.

HMM–Bayes analysis for the trajectory fragment boxed in Fig. 2a identified one diffusive state along with two active transport states, which corresponded to retrograde and anterograde transport (Fig. 2b and Supplementary Fig. 8). The retrograde transport had a lower average velocity of 2.81 ± 0.18 μm s^−1^ compared to that of the anterograde transport (5.06 ±0 .02 μm s^−1^). Cargos were often observed to come to a full stop and pause for a few seconds before continuing in the same direction (Supplementary Fig. 8).

More complicated scenarios involving cargo pausing were also observed. Region 1 in Fig. 2b showcases an example where a short pause was followed by a rapid reversal on the same track. Such reversals may involve the detachment of the opposing motors or some regulatory process rather than the tug-of-war model^39,41^. In region 2, the cargo was initially transported retrogradely. After pausing for 0.14 s, the cargo reversed direction and remarkably diffused to another microtubule that was ~100 nm away from the original one and resumed retrograde transport. While track changes may be explained by the presence of obstacles or cargo reaching microtubule termini^41–43^, the regulatory mechanism for fast bidirectional transport in live cells remains elusive^44–46^.

As a first step in future studies of axonal transport with human disease-associated mutations, we also studied single-cargo transport in human-induced neurons (iNs). Human embryonic stem (ES) cells were *trans*-differentiated into human excitatory neurons using transient expression of neurogenin 2 (Ngn2; Fig. 2c)^47^. Similar to rat DRG neurons, these iNs also exhibit robust axonal transport with comparable behaviors including the presence of stop-and-go, reversal and track-switching motions (Fig. 2c and Supplementary Fig. 8). Our results showed that there are common dynamic features of axonal transport between rat and human neurons.

### The number of dynein changes during retrograde transport

We next show that the distribution of cargo displacements can be used to determine the number of active dynein motors being used to transport cargo. Previously, pulldown experiments and electron microscope images of neuronal endosomes indicated the association of multiple dyneins^45,48^. Attempts to measure the number of dynein motors included measurement of the stall force using large lipid droplets or ~1-μm beads^49–51^ or nanoparticle-assisted optical tethering of endosomes^52^. However, the addition of a large optical trap force could have caused additional dynein motors to become engaged, and the high laser intensity required introduced large perturbations to the cells.

**Fig. 3:**
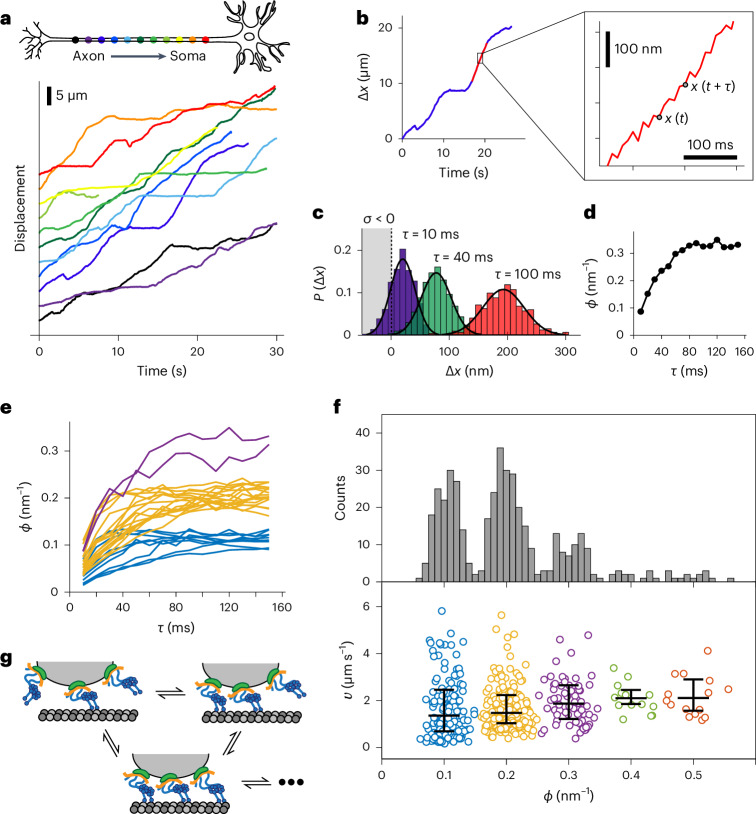
Statistical analysis of retrograde transport monitored at millisecond temporal resolution. **a**, Displacement curves of a single cargo being retrogradely transported in a rat DRG neuron, from the distal axon (dark purple) to soma (red) taken at 37 °C. The curves obtained from separate movies were displaced vertically for better visualization. **b**, Example of a constant velocity segment (red) used to measure the set of displacements $\Delta x\left(t,\tau \right)\equiv x\left(t+\tau \right)-x\left(t\right)$ for each starting time $t$ and time delay $\tau$. **c**, The probability distribution ${P}_{\tau }\left(\Delta x\right)$ for a particular displacement labeled by the subscript $n$, $\Delta {x}_{\tau }\left(t,\tau \right)={x}_{\tau }\left(t+\tau \right)-{x}_{\tau }\left(t\right)$ during different time delays $\tau =10$ ms (purple), 40 ms (green) and 100 ms (red). **d**, Relaxation curve of the entropy change per unit length $\phi =2\mu /{s}^{2}$ in equation (1). **e**, All the relaxation curves of $\phi$ obtained from the displacement curves of the same cargo are shown in **a**. The curves are classified into three clusters using *k*-means and marked with different colors. **f**, The histogram of 430 asymptotic $\phi$ values obtained from 12 single retrogradely transported cargos from 12 different neurons shows clear asymptotic quantization of the entropy change per unit length. The corresponding velocities of the constant velocity segments are also plotted. The velocities are classified into five clusters using the *k*-means clustering method. The central mark for each cluster indicates the median value, and the bottom and top lines indicate the 25th and 75th percentiles, respectively. **g**, A cartoon for our model showing how the number of independently engaged dynein motors stochastically changes during retrograde transport.

Time records of the displacement of one cargo in a DRG neuron at 37 °C, as it travels through the 900-μm microchannel, are shown in Fig. 3a. The excellent photostability of our nanoprobes enabled single-cargo tracking continuously for extended periods of time, which allowed a precise determination of the probability distribution $P\left(\Delta x\left(\tau \right)\right)$ as the cargo moved from position $x\left(t\right)$ to a new position $x\left(t+{\rm{\tau }}\right)$. For segments of roughly constant velocity (Fig. 3b, red curve), the normalized histograms $P\left(\Delta x\left(\tau \right)\right)$ were calculated at different time delays $\tau$. Figure 3c plots $P\left(\Delta x\left(\tau \right)\right)$ at $\tau$ = 10, 40 and 100 ms. The solid lines are fits to a Gaussian distribution $P\left(\Delta x\left(\tau \right)\right)$, which is fully characterized by its mean, $\mu$, and variance, ${s}^{2}$.

We examined the time dependence of a parameter, $\phi \equiv 2\mu /{s}^{2}$. As shown in Fig. 3d, the value of $\phi$ (in units of nm^−^^1^) was seen to approach an asymptotic for times $\tau$ greater than 120 ms. In about 60% of the approximately constant velocity movements, $\phi$ approached a steady-state value, and we focus our analysis on this subset of trajectories. Supplementary Fig. 9 is an example of a trajectory where $\phi$ did not reach a steady-state value, and Supplementary Fig. 10 shows the entire trajectory of a single cargo. Remarkably, the resulting curves cluster into three discrete asymptotic distributions centered at $\phi =0.1,\,0.2,\,0.3\,{\rm{n}}{{\rm{m}}}^{-1}$ (Fig. 3e).

The histogram for $\phi$ pooled from 12 different cargos also shows well-defined and equally spaced peaks with the same quantized values of $\phi$, although it is expected that there is a distribution in the diameter of each vesicle^45^. Some cargos exhibited values of $\phi$ up to 0.5 nm^−1^, whereas other cargos exhibited only the two lowest values (Fig. 3f and Supplementary Figs. 11 and 12). In sharp contrast, the corresponding velocity distribution exhibits only a modest increase in the average value with increasing $\phi$ (1:1.05:1.23 for the first three quantized values), although with large variations. Similarly, the velocity distribution of the entire trajectory of a single cargo does not exhibit quantized peaks (Supplementary Fig. 13). The statistical analysis of retrograde transport in human neurons found similar $\phi$ distributions (Supplementary Figs. 14 and 15), as expected from the high degree of structural conservation of cytoplasmic dynein among mammals.

We argue here that the quantization of steady-state $\phi$ was due to the number of active and independently tethered motor complexes. First, consider two motors attached to a vesicle that hydrolyze ATP independently and have a constant step size $\mu$. During the time when one of the motors takes a step, the other motor remains stationary, and the movement of the vesicle is half the step size, $\frac{\mu }{2}$. Because the stepping rate (number of steps per unit time) for two motors is twice as fast as with a single motor, the number of steps doubles from *n* to 2*n*, and the displacement after a given time is $n\mu ,$ which is the same as for a single motor. The derivation is supported by in vitro experiments, which showed that the average velocities of DNA origami linked to one or two dynein motors with sufficient compliance do not vary significantly, indicating nearly independent motors^7^.

Now, consider that each motor has a distribution of step sizes characterized by a variance per step, ${s}^{2}$. For any given step size in this distribution, the movement of the vesicle is one-half the step size, so the width of the distribution $s$ becomes $s/2$. In probability theory, the variance of two independent steps is additive, so the total variance with one step taken by each motor is ${s}_{2}^{2}={s}^{2}/2$. After a displacement due to 2*n* steps of two independently stepping motors, the total variance ${s}_{{\rm{total}}}^{2}$ is also additive and becomes $n{s}^{2}/2$, which is half the variance due to the displacement of one motor. Taken together, $\phi$ is doubled for two motors (see Supplementary Note 1 and Supplementary Fig. 16 for more details). In a similar manner, for cargo with $n$ active and independently tethered motors, the variance becomes $n{s}^{2}/n$. Consequently, the corresponding $\phi$ becomes $n(\frac{2\mu }{{s}^{2}})$, which is $n$-fold higher compared to the $\phi$ observed for a cargo with a single active motor. Our assignment of switching between three quantized values of $\phi$ is depicted in Fig. 3g. The deduced number of active dynein motors associated with both rat and human neuronal endosomes (average of 2.01 ± 0.05 and 2.50 ± 0.08 for rat and human neurons, respectively) is in good agreement with previous electron microscopy work and western blotting results^45,48^.

We note that while our parameter of $\phi$ represents the same experimental observable as reported in ref. ^53^, and we both observed similar quantized distributions, there are substantial differences in our work. Their quantized distributions were obtained by pooling multiple short trajectories from different vesicles, while we directly showed changes in $\phi$ for the same vesicle during the 900 μm journey. Moreover, ref. ^53^ interpreted the quantization of $\phi$ as the quantization of ‘force-producing units’ on each endosome. Although in vitro measurement has shown that the force needed to stall the motion of a bead is proportional to the number of motors^54^, we argue that the force generated to move an endosome is the drag force, which is proportional to the velocity of the vesicle.

### Stepping involves two rate-limiting steps with equal rates

**Fig. 4:**
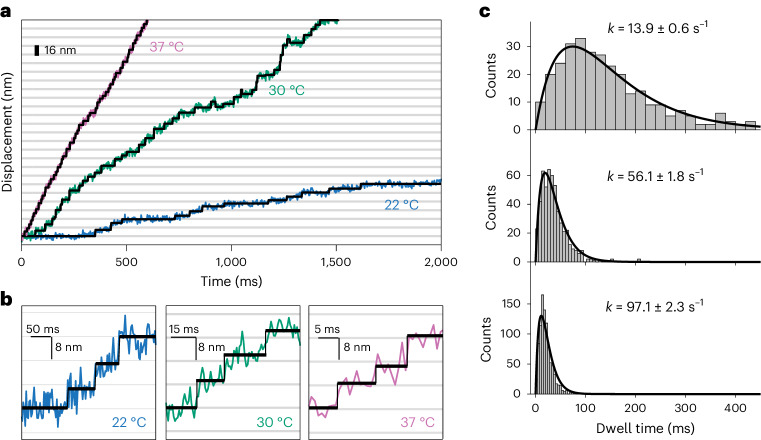
Dynein steps in live DRG neurons. **a**, Stepping traces of retrogradely transported endosomes labeled with larger probes at three different temperatures (red, 37 °C; green, 30 °C; blue, 22 °C). We synthesized and used 80 × 60 nm CSS UCNPs (NaYbF_4_:10% Gd, 8% Er@NaYbF_4_:8% Er@NaYF_4_; Supplementary Fig. 18) for experiments at 22 °C and 30 °C. Larger $160\times 90\,{\rm{nm}}$ UCNPs (Supplementary Fig. 19) were synthesized for experiments at 37 °C. The video frame rate of 1 ms consisted of a 0.8 ms integration time and 0.2 ms read-out time. **b**, Expanded views of resolved single-step time traces taken at 22 °C, 30 °C and 37 °C. Individual motor steps were identified using a step-finding algorithm (black line). **c**, The temperature-dependent histograms of dwell time between steps. The total number of recorded steps are *n* = 283, 278 and 920 steps for 22 °C, 30 °C and 37 °C, respectively. The solid lines are a convolution of two exponential functions with the same rate constants (*τk*^2^*e*^−*kτ*^, where *k* (22 °C) = 13.9 ± 0.6 s^−1^, *k* (30 °C) = 56.1 ± 1.8 s^−1^ and *k* (37 °C) = 97.1 ± 2.3 s^−1^). To be insensitive to the choice of the histogram bin size, the rate constant values (±s.e.) were determined by fitting the raw dwell times using MLE as discussed in Supplementary Fig. 22. The average time of a single step is calculated from the probability distribution $\left\langle t\right\rangle ={\int }_{0}^{\infty }{tP}\left(t\right){\mathrm{d}{t}}={\int }_{0}^{\infty }{t}^{2}{k}^{2}{e}^{-{kt}}{\mathrm{d}{t}}=2/k$ for each temperature.

Our UCNPs allowed us to visualize, for the first time, individual dynein steps in live neurons, as shown in Fig. 4 and Supplementary Fig. 17. We developed methods to synthesize larger and brighter UCNPs—6 nm (Supplementary Fig. 18) for the experiments at 22 °C and 30 °C, and $160\times 90\,{\rm{nm}}$ (Supplementary Fig. 19) for the experiments at 37 °C. For the largest UCNPs (more than 40× brighter than our 28 nm particles), about $5\times {10}^{6}$ photons per second were detected. These brighter probes allowed us to accumulate large single-step datasets for statistical analysis. However, our analysis of the data showed that we could only resolve single molecular steps at the slowest stepping rates.

The step-size histogram measured at 22 °C (Supplementary Fig. 20) displayed multimodal distribution with only partially resolved peaks at about 8 nm and larger steps. This distribution for the retrograde vesicle represents the motions of the full assembly of dynein, dynactin, cargo adapter and vesicle in live neurons and is in good agreement with in vitro measurements of purified single yeast dynein dimers at room temperature^12,55^. From this, we conclude that the 22 °C step-size histogram is the measurement of single dynein motors. Note that if one of the motor domains of a dynein motor steps by 16 nm along the microtubule and the other motor domain does not move, the vesicle will move by 8 nm. Because the microtubule-binding sites are spaced 8 nm apart, the minimum step of the dynein motor should be 4 nm (Supplementary Fig. 21).

The velocity of dynein increased by about 7× as the temperature increased to 37 °C (the internal temperature of rats and human neurons), and the average dwell time decreased markedly to about 20.6 ms (Fig. 4c). Notably, the dwell-time histograms at all three temperatures fit well with a kinetic scheme consisting of the stepping cycle described by two sequential rate constants, ${k}_{1}$ and ${k}_{2}$. The mathematics of this model is straightforward and discussed in Supplementary Fig. 22. The best fit to the data is ${k}_{1}={k}_{2}$. We stress here that fitting the dwell times to a prebinned histogram is subject to human bias because the bins could be chosen to suppress the rapidly rising portion of the dwell-time distributions. Here we directly fit dwell-time data points using maximum likelihood estimates (MLE). As shown in Supplementary Fig. 22, the uncertainties of the fits to prebinned histograms are larger when compared to fitting the dwell times.

Moreover, the Arrhenius plot (Supplementary Fig. 23) shows that the two rates are consistent with thermal activation, with an activation energy of 25.1 ± 23 kcal mol^−1^. The fact that the three dwell times are consistent with a single activation energy allows us to infer that the dwell-time distributions taken at the higher temperatures must have the same number of motors. The dwell-time distribution of a vesicle being moved by two independent motors would have half of the dwell time of a single motor. Thus, we conclude that all the single-step data taken at different temperatures and with different vesicles shown in Fig. 4 are measurements of different single motor systems moving at the low end of their allowable stepping rates.

The shape of the dwell-time distribution is further quantified by the ‘randomness parameter’^56^, defined as

1 $$r\equiv (\left\langle {\tau }^{2}\right\rangle -{\left\langle \tau \right\rangle }^{2})/{\left\langle \tau \right\rangle }^{2}.$$

It has been shown that the inverse of the randomness parameter ${r}^{\,-1}={n}_{\mathrm{min} }$ provides the lower bound of the number of kinetic steps within an enzymatic cycle^57^. At all three temperatures, we measured an ${n}_{\min } \sim 2$ (Supplementary Fig. 24), indicating that there are at least two rate-limiting steps in each cycle. In summary, within each dynein stepping cycle, there are two rate-limiting steps with the same rates that are both thermally activated.

## Discussion

The AAA+ modules on each dynein contain four ATP binding sites from AAA1 to AAA4 (ref. ^3^). The ATP binding site between the AAA1 and AAA2 domains (labeled as the ‘AAA1 site’) has been identified as the primary catalytic site^6^. AAA2 lacks the catalytic Walker B motif, so ATP hydrolysis at this site is not possible^3^. The ATPase activity at AAA3 has also been determined to be essential for dynein motility^58^. The inhibition of ATP binding in AAA4 has recently been demonstrated to halt dynein motility, showcasing its pivotal regulatory role in dynein activity^59^. However, blocking ATP hydrolysis does not substantially impact dynein motility^58^. Therefore, each stepping cycle of dynein could involve the hydrolysis of ATP at AAA1 and possibly AAA3 as well.

The predominant model for dynein stepping, the so-called ‘active-cycling’ model, requires the hydrolysis of ATP only at the AAA1 site for a single step^60^. In this model, ADP remains bound at AAA3, and ATP at AAA1 actively cycles between ATP, ADP·Pi, ADP and Apo states (Supplementary Fig. 25).

We found that the in vivo dwell-time distributions are dominated by two thermally activated rate constants. These dynamics were hidden from the in vitro studies since the measurements were taken at ATP that were approximately three orders of magnitude lower than in cells. Based on this finding and previously measured kinetic rates, the one-ATP model is incompatible with the two equal and thermally activated rate constants. In ATP hydrolysis, ${\mathrm{Apo}}\to {\mathrm{ATP}}\to {\mathrm{ADP}}\cdot {\mathrm{Pi}}\to {\mathrm{ADP}}\to {\mathrm{Apo}}$, the hydrolysis of ATP and the dissociation of ADP have been found to occur on timescales of ≤1 ms in cellular conditions at room temperature^61^. Single-molecule in vitro experiments have measured an ATP binding rate constant *k*_MT_ = 0.29 ± 0.10 μm^−1^ s^−1^ (ref. ^62^). Assuming 3 mM of ATP concentration in the axon^63–65^, the ATP binding rate is 870 s^−1^. Thus, the only possible thermally activated rate is the step ${\mathrm{ADP}}\cdot {\mathrm{Pi}}\to {\mathrm{ADP}}$. Furthermore, because the dwell-time distribution fits two sequential rate constants, we are forced to conclude that the two rate-limiting steps are the thermal desorption of two Pi’s from the AAA1 and AAA3 sites.

It has been shown that the microtubule-binding domain of dynein can be in either a strongly or weakly bound state^3,6^. During the submillisecond stepping transition (Fig. 4b), the motion of the vesicle induces an equal and opposite ‘reaction force’ (Newton’s third law) on the stepping dynein motor domain attached to the microtubule. It seems plausible that dynein should be in a strongly bound state so that it is less likely for dynein to detach from the microtubule. However, optical tweezer measurements (Supplementary Fig. 26) showed that dynein is weakly bound to the microtubule when ADP is on the AAA3 site^66^. This suggests that for dynein to transition to the strongly bound state, ADP would need to leave AAA3.

We note that the molecular details of how dynein causes the vesicle to move during the hydrolysis cycle remain unclear. A ‘power stroke’ is often postulated in the molecular motor literature^3,6^, but how the hydrolysis of ATP generates a force has not been proposed. An alternate explanation is that the dynein motion may be largely due to Brownian motion fluctuations. The microscopic molecular nature of how motors advance on microtubules remains debated^67,68^, and we plan to address this point in a subsequent publication.

The requirement that dynein hydrolyzes two ATP’s per step, as revealed by our measurement and analysis of the dwell-time distribution, led us to re-examine the experimental evidence that originally led to the active-cycling model. In Supplementary Note 2, we argue that earlier measurements are also consistent with the two-ATP model. In previous work, the average number of Pi’s generated per dynein step was calculated to be ~1.03 Pi per step^12,69^. However, because the phosphate release rate and average velocities were obtained from different experiments, the variation of these values can be large. For example, a similar analysis of the data reported in a subsequent publication^69^ results in 1.9 Pi per step.

Second, a Michaelis–Menten (MM) analysis of the dynein velocity versus ATP agrees with a Hill coefficient of $n=1$ that is expected if the activity involves the hydrolysis of a single ATP^70^. A fit to $n=2$ and a sigmoidal dependence of the enzyme kinetics on ATP requires that there must be hydrolysis of two ATPs. However, it has been shown that if the enzymatic cycle contains steps separated by effectively irreversible transitions such as the hydrolysis of ATP in live cells, the MM reaction rate can also fit with a Hill coefficient $n=1$, even if there are two or more ATP hydrolysis^71^. To demonstrate this point, we simulated the dwell-time distributions for dynein stepping involving two sequential ATP hydrolysis (Supplementary Fig. 27) and showed that the ATP-dependent velocities satisfied the MM equation with a Hill coefficient of $n=1$.

Moreover, we calculated ${n}_{\min }$ as a function of the ATP concentration from the simulations (Supplementary Fig. 27). At high ATP, where the desorption of two Pi’s is rate-limiting, ${n}_{\min } \sim 2$ as observed in the in vivo measurements. At low ATP, where the binding of two ATPs becomes rate-limiting, ${n}_{\min }$ should also be 2. At intermediate ATP, where the ATP on-rate is equal to the thermal desorption rate of Pi, ${n}_{\min }$ should become 4. Therefore, a future measurement of ${n}_{\min }$ as a function of ATP would clearly differentiate between the one-ATP active-cycling model and our proposed two-ATP model.

In in vitro studies where the rate-limiting step is the on-rate of ATP, the proposed two-ATP model predicts that the dwell-time distribution should also fit two equal but slower rate constants. We note that if the thermal desorption ${\mathrm{ADP}}\cdot {\mathrm{Pi}}\to {\mathrm{ADP}}\to {\mathrm{Apo}}$ is sequential, the binding of ATP must also be sequential. While earlier work only showed a single exponential decay, with improved time resolution, the dwell-time distribution from the most recent in vitro experiments appears to be a better fit to two equal rate constants, provided the dwell-time distribution is fit to the raw dwell times rather than prebinned histograms^10^, as discussed in Supplementary Fig. 22.

In conclusion, we described the development of substantially improved photostable UCNPs, which enabled long-term SPT in live cells with millisecond temporal resolution simultaneously with few nanometer spatial resolution. This technology was used to measure the number of active dynein motors responsible for retrograde transport in the axons of live neurons. From a statistical analysis of the displacement fluctuations made possible by long-time tracking, we found that the number of dynein motors transporting cargo varied stochastically between one and five dynein motors.

With the high brightness and photostability of UCNPs, we could measure individual dynein steps in live neurons. Two thermally activated and equal rates were obtained from the temperature-dependent dwell-time distributions, leading us to propose a model for dynein stepping that requires the hydrolysis of two ATPs during each chemomechanical cycle. This model differs from previous conclusions^60,70^ where the AAA3 site acts as a switch that allows multiple single motor steps with repeated hydrolysis occurring only at AAA1. From this data and previous measurements, we have developed a model that requires the time-ordered hydrolysis of ATP at both AAA1 and AAA3. A more detailed mechanochemical study of dynein stepping will be presented in a future publication.

## Methods

Our research complies with all relevant ethical regulations in accordance with Stanford University’s institutional guidelines.

DRG neurons were obtained from Sprague–Dawley rats (Charles River Laboratories) using an experimental procedure approved by the animal ethics committee of the Administrative Panel on Laboratory Animal Care of Stanford University (APLAC-20608), in accordance with Stanford University’s Institutional Animal Care and Use Committee policies for the use of animals in research. The Sprague–Dawley rats were ordered as timed-pregnant rats. The age of the mother rats was not specified but was in the range of 4–8 months. Each dissection used one mother rat, which had eight embryos on average. The results reported in this paper came from about ten dissections at embryonic day 18 (E18).

H1 human ES cells (WA01; RRID: CVCL_9771; National Institutes of Health (NIH) approval NIHhESC-10-0043) were obtained from WiCell Research Resources (Wicell), maintained in feeder-free conditions using mTeSR1 medium (STEMCELL Technologies) and used at intermediate (~50) passage numbers to generate iNs^72^. They were routinely analyzed for (1) characteristic hiPSC cell morphology, (2) expression of pluripotency markers OCT4, NANOG and SOX2, (3) ability to differentiate in vitro into multiple cell types and (4) a normal complement of 46 chromosomes by karyotyping (every 20 passages). These standards are consistent with established guidelines for the maintenance of human pluripotent stem cells^73,74^.

### Chemicals and reagents

All starting materials were purchased from commercial supplies. The rare-earth salts (such as YCl_3_·6H_2_O, YbCl_3_·6H_2_O, ErCl_3_·6H_2_O and TmCl_3_·6H_2_O) and other chemicals (such as NH_4_F, NaOH, sodium oleate, cyclohexane, ethanol, octadecene (>90%), oleic acid (>90%), Igepal CO-520, tetraethyl orthosilicate and ammonia solution (28%)) were purchased from Sigma-Aldrich. Streptavidin-coated QD705 (CdSe) was purchased from Thermo Fisher Scientific (Q10163MP). Silane–PEG(3.4k)–biotin was purchased from Laysan Bio. The 2-(methoxy-(polyethyleneoxy)propyl) trimethoxysilane was purchased from Gelest. The FluoReporter Cell-Surface Biotinylation Kit (F-20650) was purchased from Molecular Probes. All chemical reagents of analytical grade were used directly without any further purification.

### Nanoparticle synthesis

Detailed synthesis protocols are included in the Supplementary Methods.

### Nanoparticle characterization

X-ray diffraction measurements were performed on a Bruker Single Crystal Diffractometer D8 Venture (Mo Kα radiation, *λ* = 0.70930 Å). The size and morphology of UCNP were determined at 100 kV using a JEOL JEM-1400 TEM. The prepared samples were dispersed in cyclohexane and dropped onto the surface of a copper grid for TEM analysis. The upconversion luminescence emission spectra were recorded on an Edinburgh LFS-920 instrument, but the excitation source used an external 0–1 W adjustable 980 nm semiconductor laser (Beijing Hi-Tech Optoelectronic) with an optic fiber accessory, instead of the Xeon source in the spectrophotometer. Upconversion luminescence lifetime was measured with a phosphorescence lifetime spectrometer (Edinburgh Instruments, FSP920-C) equipped with a tunable mid-band optical parametric oscillator pulse laser as the excitation source (410–2,400 nm, 10 Hz, pulse width ≤5 ns; Vibrant 355II; Opotek). All the photoluminescence studies were carried out at room temperature.

### Silica coating of UCNPs

UCNPs were coated with silica using a reverse microemulsion method^36,75^. The protocol was modified for different starting materials of UCNPs, as shown in Supplementary Table 1. The protocol used for silica coating of the core-only NaYF_4_:20% Yb and 2% Er is described in detail here. Igepal CO-520 (1,000 mg) was added to 10 ml of cyclohexane and dispersed using sonication for 10 min. Then 200 μl of as-prepared oleic acid-capped UCNPs solution was added, and the mixture was stirred vigorously for 1 h. Subsequently, 150 μl of ammonia solution (28%) was added, and the mixture was stirred overnight until a transparent emulsion was formed. Tetraethyl orthosilicate (12 μl) was then added, and the mixture was gently stirred for 2 days. The entire process was carried out at room temperature. Silica-coated UCNPs were precipitated by adding 10 ml of ethanol and collected by centrifugation (21,000*g*, 20 min). The pellet was dispersed in 40 ml of ethanol by sonication and then collected by centrifugation (21,000*g*, 20 min). The washing step was repeated, and the purified nanoparticles were redispersed in 3 ml of ethanol for storage.

### Surface modification of silica-coated UCNPs

To incorporate PEG onto silica-coated UCNPs, 20 mg of 2-(methoxy-(polyethyleneoxy)propyl) trimethoxysilane (silane-mPEG; molar mass ~500 g mol^−1^) was dissolved in 3 ml of ethanol and dropwise added to silica-coated UCNPs in ethanol (1 ml) while stirring. For biotin-conjugated UCNPs, silane–PEG(3.4k)–biotin (4 mg) was dissolved in 500 μl of ethanol and added to the mixture while stirring. Subsequently, 200 μl of water and 10 μl of ammonia (28%) were added to the mixture. The solution was heated to 50 °C and stirred overnight. The PEGylated UCNPs were then cooled to room temperature. The solution was diluted to 10 ml in ethanol and collected by centrifugation (16,900*g*, 20 min). The UCNPs were washed twice in ethanol and twice in potassium phosphate buffer (PBS, pH 7.4) to remove excess reagents. The particles were finally filtered through a 0.22 μm filter and stored in PBS buffer at 4 °C.

### Binding assay of biotin–UCNPs and streptavidin–QDs

To test the specific binding of biotinylated UCNPs (btn-UCNPs), 5 μl of 40 pM btn-UCNPs or UCNP@SiO_2_-mPEG was sonicated and incubated with 10 μl of 20 nM streptavidin-coated QD705. After 5 h of incubation, the mixture was diluted 10× and drop cast on TEM grids for TEM imaging.

### Single-particle binding assay on biotinylated PEG coverslips

To test the specific binding of btn-UCNPs, we also performed a single-particle binding assay on PEGylated coverslips. PEGylated coverslips and sample chambers were prepared following the previous protocol^76^. Ten microliters of NeutrAvidin in PBS (1 μM) were added to the sample chamber and incubated for 5 min. Unbound NeutrAvidin was washed thoroughly with 100–200 μl of PBS buffer. Btn-UCNPs (30 pM) were injected into the sample chamber and incubated for 5 min before being washed thoroughly. For control experiments, NeutrAvidin was not added before btn-UCNPs incubation.

### Cell-surface binding assay

To examine the specific binding and nonspecific sticking of btn-UCNPs on the cell surface, we biotinylated the cell surface of Hela cells and performed binding experiments similar to what was done on PEGylated coverslips. Hela cells were plated in a Lab-Tek eight-well chamber at a density of 30,000 cells per well and cultured with DMEM (high glucose), 10% fetal bovine serum (FBS), 2 mM l-glutamine and 100 U ml^−1^ penicillin–streptomycin. Cells were grown to 95% confluent before the experiment. The stock biotinylation kit containing biotin-XX sulfosuccinimidyl ester (0.2 mg ml^−1^ in DMSO) was diluted to 5 μg ml^−1^ in a culture medium. The culture medium in the Lab-Tek well was aspirated, and 150 μl of 5 μg ml−1 biotinylation reagent was added. The cells were incubated for 15 min at room temperature. Subsequently, the cells were washed with cold PBS three times to remove unreacted reagents. A total of 100 μl of NeutrAvidin in PBS (1 μM) was added to the cells, and the cells were then incubated for 10 min at room temperature. Unbound NeutrAvidin was washed thoroughly with cold PBS five times. Furthermore, 100 μl of btn-UCNPs (100 pM) were then added to the cells and, the cells were then incubated for 5 min at room temperature. Unbound UCNPs were washed thoroughly with cold PBS five times. For control experiments, NeutrAvidin was not added.

### Rat DRG neuron culture

DRG neurons were dissected out of Sprague–Dawley rat embryos at E18 in Hanks’ buffered saline solution. The DRG cells were then treated with 5 ml of 0.25% trypsin for 30 min at 37 °C followed by mechanical trituration. An equal volume of Dulbecco’s modified Eagle’s medium (+10% FBS) was added to quench trypsin, and the dissociated cells were spun down at 200 r.p.m. for 8 min. Cells were resuspended in DRG maintenance media (neurobasal, B27 plus, 2 mM l-glutamin/GlutaMAX, 100 U ml^−1^ penicillin–streptomycin and 50 ng ml^−1^ nerve growth factor). After counting the cell density, cells were spun down and resuspended in an appropriate volume of maintenance media to reach a final concentration of 10 million cells per ml. Ten microliters of cells were plated in the soma compartment of the microfluidic device. The microfluidic device (SND900 from Xona Microfluidics) was pre-assembled on 24 mm × 60 mm No. 1.5 coverslips precoated with 0.5 mg ml^−1^ poly-d-lysine overnight. Cells were maintained at 37 °C with 5% CO_2_ for up to 21 days. On 2 days in vitro (DIV 2), the media was replaced with antimitotic media (maintenance media containing 1 μM cytosine arabinoside) to suppress glial cell proliferation. The media was replaced back to normal maintenance media on DIV 3. Rats used in this study were treated per Stanford University’s institutional guidelines.

### Human-induced neuron

iNs were generated from *trans*-differentiating human ES cells (WA01/H1 cell line, NIH registry 0043) as described in ref. ^47^. In brief, H1 cells were plated on a matrigel-coated surface and transduced lentivirally to express the single transcription factor Ngn2 and the selection marker puromycin N-acetyl transferase (PuroR) in a tetracycline-inducible fashion, driven by tetracycline operator tetO promoter plus the co-expression of transactivator. After lentiviral infection (day 1), doxycycline (2 mg l^−1^) was added on day 0 for 3 days, and puromycin (1 mg l^−1^) selection was performed on days 1 and 2. On day 3, differentiating human iN cells were detached, plated on one compartment of a matrigel-coated microfluidic chamber (10 μl of 10 million cells per ml) and maintained in Neurobasal A medium with B27 supplement.

### UCNPs internalization in neurons

For both rat DRG and human iNs, live-cell imaging was done between DIV 14 and DIV 21. Media in the axon or soma chambers (for retrograde or anterograde transport, respectively) were removed, and 10 μl of 1 μM of biotin–WGA (Vector Laboratories, B-1025) was added. After 5 min incubation, 150 μl of maintenance media was used to wash away free biotin–WGA. Subsequently, 10 μl of 1 μM of streptavidin was added for 3 min. Excess streptavidin was removed by washing with 150 μl of maintenance media twice. After washing, 1 nM of biotin–UCNPs (diluted in maintenance media) was added, and the cells were incubated at 37 °C with 5 % CO_2_ for 2 h. Before imaging, free UCNPs were washed away with 150 μl of maintenance media.

### Live-neuron imaging

Imaging was performed using a home-built microscope with wide-field epi-illumination of a 976 nm fiber laser through a ×100 oil-immersion objective (numerical aperture (NA) = 1.49; Nikon). A microscope stage-top incubator (STRF-WELSX-SET; Tokai Hit) was used for live-cell imaging at 37 °C and 5% CO_2_. The upconverting luminescence signal was recorded on an electron multiplying charge-coupled device camera (iXon 897; Andor) using Andor Solis software. To achieve sufficient spatial resolution at 10 ms exposure time for fluctuation theory analysis, live-neuron imaging was performed at 6.5 kW cm^−^^2^. However, 976 nm excitation with much lower power density could be used to image single cargo at longer exposure times. Because there is no cell autofluorescence, we were able to achieve the theoretical spatial localization precision $\delta x \sim (\lambda /2\,{\mathrm{NA}})/\sqrt{n}$, where *λ* is the wavelength of the detected light, NA is the numerical aperture of the microscope objective and $n$ is number of detected photons is achieved. For the experiments shown in Fig. 2, $n=1,000,\,\delta x \sim 6.4\,{\mathrm{nm}}$. For the step-resolved experiments at 37 °C shown in Fig. 4, $n=5,000,\,\delta x \sim 2.9\,{\mathrm{nm}}$.

### UCNP saturation curve measurement

Procedures for measuring UCNP saturation curves were established previously^22^. Approximately 400 ng ml^−1^ UCNPs in cyclohexane were drop cast onto a clean and dry No. 1.5 cover glass precoated with 1% (wt/vol) poly-l-lysine. Cyclohexane was used to rinse off excess nanoparticles after 1 min incubation. For rigid support, the cover glass was attached to a standard microscope slide using double-sided tape. Custom IDL code was used to identify individual point spread functions and perform a 2D Gaussian fit to determine the upconversion emission rate. To correct measured UCNP luminescence by the wide-field illumination profile, the microscope stage was scanned across the field of view with an equal step size of 1 µm. The position-dependent luminescence profile of a single UCNP was used to compute the wide-field illumination profile.

### Correlative scanning electron microscope (SEM) and wide-field fluorescence imaging

Nanoparticles were drop cast as described above onto a glass coverslip with an alphanumerically labeled grid pattern marked in 50 µm increments (IBIDI grid-50, IBIDI). The sample was first characterized under wide-field optical illumination. Subsequently, a thin layer of 2 nm gold-palladium was sputter-coated (Denton Vacuum) onto the same sample to enhance conductivity, and nanoparticles were imaged using a Zeiss Sigma field emission SEM (Carl Zeiss Microscopy) and InLens secondary electron detection. The fine grid pattern served as a navigation guide to locate the field of views that had previously been optically characterized. Once registration was established between the geometric patterns of the fluorescence image and electron micrograph, we zoomed in to verify the oligomeric state and the size of each individual nanoparticle.

### SPT

For SPT, individual point spread functions were localized and their time trajectories were resolved using custom MATLAB scripts which performed 2D Gaussian fitting with the multiple-target tracing method^77,78^.

### HMM–Bayes analysis

Single-cargo trajectories were analyzed with HMM–Bayes, which infers stochastic switching between diffusive and directed transport states from measured particle displacement^40^. HMM–Bayes software written in MATLAB was downloaded from http://hmm-bayes.org/ and used with default parameters. The program automatically annotates when and where each type of motion occurs in space and time along the trajectory with single-step resolution.

### Dynein step size determination

To determine the molecular step size in live DRG neurons, retrograde transport was imaged at 2.5 ms exposure time at 22 °C and 30 °C and at 1 ms frame rate (0.8 ms integration time and 0.2 ms data transfer time) at 37 °C. Single-particle trajectories were obtained as described above. Step sizes were determined by a step-finding program developed by Kerssemakers et al.^79^. Step sizes smaller than the experimental noise of 4 nm were excluded.

No statistical methods were used to predetermine sample sizes, but our sample sizes are similar to those reported in previous publications (refs. ^12,22,28,38,54^).

### Reporting summary

Further information on research design is available in the Nature Portfolio Reporting Summary linked to this article.

## Online content

Any methods, additional references, Nature Portfolio reporting summaries, source data, extended data, supplementary information, acknowledgements, peer review information; details of author contributions and competing interests; and statements of data and code availability are available at 10.1038/s41589-024-01694-2.

## Supplementary information

Supplementary Information Supplementary Notes 1 and 2, Methods, Figs. 1–27 and Table 1.

Reporting Summary

## Data Availability

All data that support the conclusions are available from the authors on request.

## Code availability

The code used in this paper is available from the corresponding author upon request.
