# Supplementary material for: Nanometer-resolution tracking of single cargo reveals dynein motor mechanisms
Source: Nat Chem Biol. 2024 Aug 1;21(5):648–56. doi: 10.1038/s41589-024-01694-2 (PMC11785820; doi:10.1038/s41589-024-01694-2)
Supplement: Supplementary file 1 — Supplementary Notes 1 and 2, Methods, Figs. 1–27 and Table 1. [file 41589_2024_1694_MOESM1_ESM.pdf]

nature chemical biology

Supplementary information https://doi.org/10.1038/s41589-024-01694-2

Nanometer-resolution tracking of single cargo reveals dynein motor mechanisms

In the format provided by the authors and unedited

Table of Contents

Supplementary Notes	2
Supplementary Methods	6
Supplementary Information Figures	9
Supplementary Fig. 1. Photostability of UCNPs	9
Supplementary Fig. 2. TEM image of the core-only $NaYF_4$: 20%Yb, 2% Er	10
Supplementary Fig. 3. XRD patterns of the UCNPs.	11
Supplementary Fig. 4. TEM images of streptavidin coated quantum dots with biotinylated UCNPs	12
Supplementary Fig. 5. Wide-field fluorescent images of UCNPs	13
Supplementary Fig. 6. Live-cell imaging of btn-UCNPs on HeLa cells	14
Supplementary Fig. 7. TEM image of the core-shell-shell $NaYF_4$@ $NaYbF_4$:8% Er@$NaYF_4$.	15
Supplementary Fig. 8. HMM-Bayes analysis of the transport in rat DRG and human iNs	16
Supplementary Fig. 9. One example of segment where ϕ does not reach a steady-state value	17
Supplementary Fig. 10. Displacement segments with different ϕ values during the transport	18
Supplementary Fig. 11. ϕ curves for twelve individual retrograde endosomes in DRG neurons.	19
Supplementary Fig. 12. Pooled effective entropy ϕ curves from 12 different retrograde cargoes.	20
Supplementary Fig. 13. Velocity distribution of single retrogradely transported cargo	21
Supplementary Fig. 14. ϕ curves for eight individual retrograde endosomes in human iNs.	22
Supplementary Fig. 15. ϕ distributions for rat DRG neurons and human iNs.	23
Supplementary Fig. 16. Simulation of two dynein motors stepping	24
Supplementary Fig. 17. More retrograde transport step-size traces	25
Supplementary Fig. 18. TEM images of larger UCNPs for step-resolving experiments	26
Supplementary Fig. 19. TEM images of larger UCNPs for step-resolving experiments	27
Supplementary Fig. 20. Comparison of dynein step size distributions in live neuron and in vitro	28
Supplementary Fig. 21. Simulation of one dynein motor stepping	29
Supplementary Fig. 22. Temperature-dependent dwell-time histograms of dynein stepping	31
Supplementary Fig. 23. Arrhenius plot of the dynein stepping rate constants	32
Supplementary Fig. 24. n_{min} values	33
Supplementary Fig. 25. Previous single molecule force measurements	34
Supplementary Fig. 26. Schematic of the previously proposed active cycling-model	35
Supplementary Fig. 27. Simulated dwell-time distributions based on the 2-ATP model.	36
Supplementary Tables	37
Supplementary References	38

Supplementary Notes

Supplementary Note 1. The variance of displacement due to multiple steps

The cumulative variance for a two-motor vesicle after many steps is reduced by a factor of 2 when compared to a single motor. This analytic result is further supported by simulations shown in Supplementary Fig. 16.

To see this, consider the displacement of a vesicle due to two independent but identical motors. "Independently but identical motors" is defined to mean that the anchor points of the motors to the vesicle have sufficient elastic compliance so that the movement of the stepping motor domain does not force the non-stepping motor domain to detach from the microtubule. Under this assumption, the motion of the vesicle is the difference between the stepping and non-stepping motors. In the case of 2 dynein motors consisting of four dynein motor domains, if the two motors are attached to different points on the same vesicle, we believe there is enough flexibility in the system that allows the non-stepping motor to remain attached to the microtubule since the anchor points of the motor complexes to the vesicles are not complexly rigid. Similarly, in Derr, N. D., et al.[1], there is sufficient compliance in the attachment points of the dynein to the DNA scaffold so that each dynein motor can be modeled as independent.

However, in the case of 4 dynein motor domains attached to the same dynactin, such as in the DDR complex[2], whose velocity is faster than the DDB complex consisting of 2 dynein motor domains[2]. We believe this observation is consistent with our view that if the attachment of the non-stepping motor domain is sufficiently rigid, some fraction of the steps will cause one or more of the non-stepping ones to detach, slip forward and then reattach. Thus, naively, we would expect that $\phi = 2\mu/s^2$ for a single DDR motor would have a slightly higher mean displacement μ and half of the statistical fluctuations compared to one DDB motor. However, in our live cell observations, we do not know the fraction of dynein motors in the DDB and DDR configurations.

The probability distribution $P\big(\Delta x(\tau)\big)$ as the cargo moved from position $x(t)$ to a new position $x(t+\tau) = x(t) + \Delta x(\tau)$. For segments of roughly constant velocity (red curve in Fig. 3b), the normalized histograms $P\big(\Delta x(\tau)\big)$ were calculated at different time delays τ. Fig. 3c plots $P\big(\Delta x(\tau)\big)$ at τ = 10, 40, and 100 ms. The solid lines are fits to a Gaussian distribution

$$P\big(\Delta x(\tau)\big) = \frac{1}{\sqrt{2\pi}s} e^{-\frac{(\Delta x(\tau)-\mu)^2}{2s^2}}, \tag{1}$$

where μ and $s^2 = \langle x^2 \rangle - \mu^2$ are the mean and variance of the distribution. As discussed in the main text, the stepping rate for 2 motors is twice as fast as with a single motor, but each step moves the vesicle half the distance the number of steps doubles from N to $2N$, but the displacement after a given time is $(2N)\left(\frac{\mu}{2}\right) = N\mu$ remains the same.

In probability theory, the variance of two independent steps is additive, so the total variance with one step taken by each motor is $s_2^2 = (s/2)^2 + (s/2)^2 = s^2/2$. After a displacement due to $2N$ steps of the two motors, the variance becomes half the variance in displacement for one motor: $(2N)(s/2)^2 = Ns^2/2$,. Taken together, ϕ is doubled for 2 motors.

Similarly, for a cargo with n active and independently tethered motors, following a displacement of $N\mu$ due to nN steps, the variance becomes $(nN)(s/n)^2 = Ns^2/n$. Consequently, the corresponding

ϕ can be expressed as $\phi = \frac{2\,N\mu}{N\,s^2/n} = n\frac{2\mu}{s^2}$, which is n-fold higher compared to the ϕ observed for a cargo with a single active motor.

More quantitatively, assume the probability distribution of a displacement after the ith step for each motor is $p_i(x_i)$ for a vesicle. After each step, the vesicle movement is the average of the stepping and the non-stepping motor. Hence, the motion of the vesicle is $x_i/2$, where x_i is the movement due to a single motor attached to the vesicle. Similarly, the dispersion s_i in $p_i(x_i)$ is also reduced, $s_i \rightarrow s_i/2$ as argued in the Main Text. After N steps, the displacement of the vesicle is $p_1(x_1) * p_2(x_2) * \ldots * p_N(x_N)$.

For simplicity, consider the case where the single-step distribution is approximated as a Gaussian with a mean μ_f and a dispersion s_f^2,

$$f(x) = \frac{1}{\sqrt{2\pi}s_f}\exp\left[-\left(x-\mu_f\right)^2/2s_f^2\right]. \quad (2)$$

In general, after multiple convolutions, the Central Limit theorem says the convolution of many discrete integrable single-step distributions is well-approximated by continuous Gaussian distribution, so this assumption is much less than appears.

The distribution of a cargo driven by two motors is the convolution of two distributions $f(x) * g(x)$. In the case of two Gaussian functions, it can be shown that[3],

$$\begin{aligned} f(x) * g(x) &= F^{-1}\left[F\left(f(x)\right)F\left(g(x)\right)\right] \\ &= \frac{1}{\sqrt{2\pi\left(s_f^2+s_g^2\right)}}\exp\left[-\left(x-\left(\mu_f+\mu_g\right)\right)^2/(2\left(s_f^2+s_g^2\right)\right] \end{aligned} \quad (3)$$

where

$$F\left(f(x)\right) = \int_{-\infty}^{\infty} f(x)e^{-2\pi ikx}dx \quad (4)$$

is the Fourier transform of $f(x)$, and

$$F^{-1}\left(f(k)\right) = \int_{-\infty}^{\infty} F(k)e^{+2\pi ikx}dk \quad (5)$$

is the inverse Fourier transform. For two identical motors, $\mu_f = \mu_g = \mu/2$, where μ is the average motion when only one motor is operational. After a period of time and $2N$ steps, the displacement of a vesicle with two active motors is $p_1(x) * p_1(x) * \ldots * p_N(x) \propto \exp[-(x-2N\mu)^2/(2N(s/2)^2)]$. Thus, the cumulative variance for a two-motor vesicle is reduced by a factor of 2 when compared to a single motor. This analytic result is further supported by simulations shown in Supplementary Fig. 16.

In the case of two dynein motors attached to the vesicle at different points, after multiple steps, the attachments of the two motors to the microtubule will separate due to the stochastic nature of the stepping motors. When this separation approaches the diameter of the vesicle, the opposing forces exerted on the two motor microtubule attachment points will become significant. At this point, we

conjecture that one of the two motors will momentarily detach from the microtubule, and then reattach to relieve the stress. It has been shown that the "forward tension" force needed to detach the trailing dynein exerted by the leading dynein taking a step is considerably less than the "backward tension" force generated by the trailing dynein stepping forward[4,5]. Hence, the trailing motor is more likely to separate and reattach closer to the leading dynein motor. In this scenario, the vesicle will appear to take a large step forward. When averaged over many steps, the average displacement should be slightly larger than that on a single motor. This reasoning is consistent with the slight increase in the average velocity with increasing ϕ.

Supplementary Note 2. Analysis of evidence in support of the active cycling model
There are several arguments in the literature used to support of the active-cycling model. However, upon closer inspection, these arguments do not provide compelling evidence this model.

(i) A bulk assay was used to measure the rate of Pi generation due to ATP hydrolysis per dynein stepping under saturating concentrations of microtubules binding to dynein[6]. The authors argue that for an ATPase rate of $k_{cat}{\sim}16\ Pi$ per second per dimer, if the step is $\sim 8\ nm$ steps with a 0.8 probability of moving forward and a velocity of $100\ nm \cdot s^{-1}$ equates to $1.026\ Pi$ per step[6].

In a detailed reanalysis of Ref. [6], we find that their data is more consistent with $2\ Pi$'s generated per dynein step. However, since the phosphate release rate and average velocities were obtained from different experiments, the variation of these values can be large. For example, a subsequent publication[7] by the same group reported $20\ Pi \cdot s^{-1}$ at a velocity of $69 \pm 22\ nm \cdot s^{-1}$, which gives $1.9\ Pi$ per step.

We assert that the assignment of the average step size should account for the smaller steps that are hidden in the noise of the measurement. The minimum step sizes of a dynein motor domain are $0\ nm$ and $\pm\ 8\ nm$, corresponding to a minimum displacement of the cargo of $0\ nm$ and $\pm\ 4\ nm$. In a beautiful study of live cell tracking of dynein with very high temporal and spatial resolution [8], $\pm 4\ nm$ and $\pm\ 8\ nm$ step size intervals were clearly resolved, as shown in their Supporting Information Figs 4B, 4C [8] (see also our Supplementary Fig. 21).

In earlier reported step-size histograms[13], these small steps are not detected but $ATPs$ are still hydrolyzed during these steps. We have measured step-size distributions taken at 22°C, 30°C and 37°C shown in Fig. 5A of our *bioRxiv* paper [9]. The step-size distributions narrowed with increasing temperature, but all the distributions remained centered at $5\ nm$. The observed distributions were shown to be quantitative agreement with the reduced random-walk search time of a dynein motor domain before stepping [9]. Based on this analysis, the true step-size histogram can be approximated by a Gaussian envelope where the mean displacement is about $5\ nm$. Using $5\ nm$ as the average step size, we find that the $(Pi \cdot nm^{-1}$ per dynein$)(5\ nm) \Rightarrow 2.4\ Pi's$ per step.

(ii) Michaelis-Menten (MM) analysis of the dynein activity (velocity versus $[ATP]$) agrees with a Hill coefficient of $n = 1$ that is expected if the activity involves the hydrolysis of a single ATP[5]. A fit to $n = 2$ and a sigmoidal dependence of the enzyme kinetics on $[ATP]$ requires that there must be hydrolysis of two $ATPs$. However, it has been shown that if the enzymatic cycle contains steps separated by effectively irreversible transitions such as the hydrolysis of ATP in live cells, the MM

reaction rate can also be fit with a $n = 1$, even if there are two or more ATP hydrolysis[10].

To demonstrate this point, we simulated the dwell-time distributions for dynein stepping involving 2 sequential ATP hydrolysis. Indeed, the resulting $[ATP]$-dependent velocities satisfied the Michaelis-Menten equation with a Hill coefficient of $n = 1$. In Supplementary Fig. 27, we explicitly show that if a single dynein step involves sequential hydrolysis of two ATPs, the observed $[ATP]$-dependent velocities reported in Ref. 5 satisfies the Michaelis-Menten equation with a Hill coefficient of $n = 1$. While we also note that another earlier velocity dependence was described by to two different Michaelis-Menten parameters, which suggests two ATP binding sites[11], the primary conclusion here is that Hill coefficient analysis is inconclusive.

(iii) In a series of experiments that analyzed transient pauses in motility induced by adding ATPγS, a slowly hydrolyzing ATP analog in wild-type (WT) and mutant forms of dynein [5], the authors concluded that AAA3 domain acts as a stepping gate. Their WT homodimer motility in the presence of ATPγS fits to MM Hill coefficient of $n = 2$. In the active-cycling model, each motor domain contains one binding site, and *if* both dynein motor domains are required to be active for motility, $n = 2$ would be expected. The group also studied the activity of an SRS-WT heterodimer, where movement can only be generated by the dynein motor domain, and their data was consistent with $n = 1$. While suggestive, the motility of the mutant construct was compromised and there is no guarantee that the enzymatic activity reflects dynamics of the WT-homodimer. Furthermore, the data in Ref. [5] as pointed out above in (ii) does not rule out a model that demands the hydrolysis of 2 ATP's on the active dynein motor domain.

Supplementary Methods

Nanoparticle synthesis

Synthesis of 22 nm $NaYF_4:Yb_{0.2}Er_{0.02}$ used in single-particle binding assays: This type of UCNPs were synthesized and reported in our previous publication[12]**.** Given amounts of $YCl_3·6H_2O$ (0.78 mmol), $YbCl_3·6H_2O$ (0.20 mmol) and $ErCl_3·6H_2O$ (0.02 mmol) were added into a 100 mL three-necked flask with 6 mL oleic acid (OA) and 15 mL 1-octadecene (ODE) inside. The mixture was heated to 160 $°C$ to form a clear solution, then cooled down to room temperature. Two milliliters of methanol solution containing NaOH (2.5 mmol) and NH_4F (4 mmol) was added. The mixture was stirred for 30 min at room temperature, and then heated to 120 $°C$ and kept for another 30 min. Subsequently, the solution was heated to 300 $°C$ and maintained for 1 h in an argon atmosphere. After the solution was cooled naturally, 25 mL ethanol was added, and the resulting mixture was centrifugally separated (17371 g for 10 min) to a compact pellet, and the supernatant was discarded. The products were collected and washed with cyclohexane and ethanol (40 mL, 1:1, v/v) three times. The UCNPs (diameter 22.9 nm ± 0.9 nm) was stored in 3 mL cyclohexane.

Synthesis of 29 nm core-shell-shell $NaYF_4$@$NaYbF_4$: 8% Er@$NaYF_4$ used in axonal transport experiments: The CSS $NaYF_4$@$NaYbF_4$: 8% Er@$NaYF_4$ UCNPs were synthesized in our previous report[12]. Synthesis of 6.0 nm β-$NaYF_4$ seed: 1.0 mmol $YCl_3·6H_2O$ aqueous solution was added into the mixture of 10 mL oleic acid and 10 mL octadecene, then the mixture was heated to 160 ºC and kept for 30 min to remove water. The solution is cooled to < 50º C, and 0.34 g NH_4F and 2.03 g sodium oleate were quickly added into the reaction. The solution was heated to 120 ºC and kept for 30 min, and then heated to 300 ºC and maintained for 30 min at argon atmosphere. The subsequent purification steps are the same as used for 22 nm $NaYF_4$: 20% Yb, 2% Er UCNPs. The product was stored in 20 mL cyclohexane. The final particle diameter was 6.0 nm ± 0.6 nm. Synthesis of $NaYF_4$@ $NaYbF_4$: 8% Er : 0.40 mmol $RECl_3·6H_2O$ (92% Yb^{3+}, 8% Er^{3+}) was added into 3 mL oleic acid and 8 mL octadecene in a 100 mL three-neck flask. The solution was heated to 160 °C to form a clear solution under argon atmosphere. After the mixture was cooled to room temperature, 2 mL of the prepared 6.0 nm β-$NaYF_4$ seed in cyclohexane and 5 mL methanol solution containing 1 mmol NaOH and 1.5 mmol NH_4F were added into the reaction flask and stirred for 30 min. The solution was heated to 120 °C to remove low-boiling solvents for 30 min, and then heated to 300 °C and maintained for 1 h under argon atmosphere. The subsequent purification steps are the same as used for 22 nm $NaYF_4:Yb_{0.2}Er_{0.02}$. The product was stored in 8 mL cyclohexane. Synthesis of $NaYF_4$@ $NaYbF_4$: 8% Er@ $NaYF_4$: to a 100 mL three-necked flask, 3 mL oleic acid (OA) and 8 mL 1-octadecene (ODE) were added given amounts of $YCl_3·6H_2O$ (0.40 mmol). The mixture was heated to 160 °C to form a clear solution under argon atmosphere. After cooling to room temperature, half volume (4 mL) of the previously prepared $NaYF_4$@ $NaYbF_4$: 8% Er UCNPs in cyclohexane and 5 mL methanol solution containing 1 mmol NaOH and 1.5 mmol NH_4F were added into the reaction flask and stirred for 30 min. The solution was heated to remove low-boiling solvent, kept at 120 °C for 30 min, and then heated to 300 °C and maintained for 1 h under argon atmosphere. The subsequent purification steps are the same as used for the 22 nm $NaYF_4:Yb_{0.2}Er_{0.02}$. The product was stored in 8 mL cyclohexane.

Synthesis of 66 nm $NaYbF_4$: 10% Gd, 8% Er@$NaYbF_4$: 8% Er@$NaYF_4$ used for step-size measurements: Given amounts of $YCl_3·6H_2O$ (0.82 mmol), $GdCl_3·6H_2O$ (0.10 mmol) and $ErCl_3·6H_2O$ (0.08 mmol) were added into a 100 mL three-necked flask with 6 mL oleic acid (OA) and 15 mL 1-

octadecene (ODE) inside. The mixture was heated to 160 °C to form a clear solution, then cooled down to room temperature and 2 mL of methanol solution containing NaOH (2.5 mmol) and NH_4F (4 mmol) were added. The mixture was stirred for 30 min at room temperature, then heated to 120 °C and kept for another 30 min. Subsequently, the solution was heated to 300 °C and maintained for 2 h in an argon atmosphere. After the solution was cooled naturally, 25 mL ethanol was added, and the resulting mixture was centrifugally separated (12000 rpm for 10 min) to a compact pellet, and the supernatant was discarded. The products were collected and washed with cyclohexane and ethanol (40 mL, 1:1, v/v) three times. The $NaYbF_4$: 10% Gd, 8% Er (diameter of 46.7 nm ± 1.0 nm) was stored in 3 mL cyclohexane. Gd^{3+} doping was used for controlling size distribution and obtaining uniform UCNPs.

$NaYbF_4$: 10% Gd, 8% Er@$NaYbF_4$: 8% Er was synthesized using the following procedure: 0.40 mmol $RECl_3{\cdot}6H_2O$ (92% Yb^{3+}, 8% Er^{3+}) was added into 3 mL oleic acid and 8 mL octadecene in a 100 mL three-neck flask. The solution was heated to 160 °C to form a clear solution. After the mixture was cooled to room temperature, the prepared $NaYbF_4$: 10% Gd, 8% Er in cyclohexane and 2 mL methanol solution containing 1 mmol NaOH and 1.5 mmol NH_4F were added into the reaction flask and stirred for 30 min. The solution was heated to 120 °C to remove low-boiling solvents for 30 min, and then heated to 300 °C and maintained for 1 hour under argon atmosphere. The subsequent purification steps are the same as used for $NaYF_4$: 20% Yb, 2% Er. The $NaYbF_4$: 10% Gd, 8% Er@$NaYbF_4$: 8% Er (diameter of 55.2 nm ± 1.8 nm) was stored in 3 mL cyclohexane.

The inactive $NaYF_4$ layer was synthesized following a similar procedure with that of $NaYbF_4$: 10% Gd, 8% Er@$NaYbF_4$: 8% Er. 0.40 mmol $YCl_3{\cdot}6H_2O$ was used instead of 0.40 mmol $RECl_3{\cdot}6H_2O$ (92% Yb^{3+}, 8% Er^{3+}), and $NaYbF_4$: 10% Gd, 8% Er@$NaYbF_4$: 8% Er was used instead of $NaYbF_4$: 10% Gd, 8% Er. The prepared $NaYbF_4$: 10% Gd, 8% Er@$NaYbF_4$: 8% Er@$NaYF_4$ (diameter of 65.2 nm ± 1.9 nm) was stored in 3 mL cyclohexane.

Synthesis of 160 nm $NaYbF_4$: 10% Gd, 8% Er@$NaYbF_4$: 8% Er@$NaYbF_4$: 8% Er@$NaYF_4$ used for step-size measurements:

$NaYbF_4$: 10% Gd, 8% Er: Given amounts of $YbCl_3{\cdot}6H_2O$ (0.82 mmol), $GdCl_3{\cdot}6H_2O$ (0.10 mmol) and $ErCl_3{\cdot}6H_2O$ (0.08 mmol) were added into a 100 mL three-necked flask with 4 mL oleic acid (OA) and 10 mL 1-octadecene (ODE) inside. The mixture was heated to 160 ºC to form a clear solution, then cooled down to room temperature and 2 mL of methanol solution containing NaOH (2.5 mmol) and NH_4F (4 mmol) were added. The mixture was stirred for 30 min at room temperature, then heated to 120 ºC and kept for another 30 min. Subsequently, the solution was heated to 300 ºC and maintained for 2 h in an argon atmosphere. After the solution was cooled naturally, 25 mL ethanol was added, and the resulting mixture was centrifugally separated (12000 rpm for 10 min) to a compact pellet, and the supernatant was discarded. The products were collected and washed with cyclohexane and ethanol (40 mL, 1:1, v/v) three times. The resulting $NaYbF_4$: 10% Gd, 8% Er nanocrystals were stored in 3 mL cyclohexane. The nanoparticles are hexagonal prisms with diameter of ~80 nm and height of 60 nm. Gd^{3+} doping was used for controlling size distribution and obtaining uniform UCNPs.

$NaYbF_4$: 10% Gd, 8% Er@$NaYbF_4$: 8% Er: 0.40 mmol $RECl_3{\cdot}6H_2O$ (92% Yb^{3+}, 8% Er^{3+}) was added into 3 mL oleic acid and 8 mL octadecene in a 100 mL three-neck flask. The solution was heated to 160 °C to form a clear solution. After the mixture was cooled to room temperature, 1.5 mL of the prepared $NaYbF_4$: $Gd_{0.1}$, $Er_{0.08}$ in cyclohexane and 2 mL methanol solution containing 1 mmol NaOH and 1.5 mmol NH_4F were added into the reaction flask and stirred for 30 min. The solution was heated to 120 °C to remove low-boiling solvents for 30 min, and then heated to 300 °C and maintained for 1 hour under

argon atmosphere. The subsequent purification steps were the same as used for $NaY_{0.78}F_4:Yb_{0.2}Er_{0.02}$. The resulting $NaYbF_4$: 10% Gd, 8% Er@$NaYbF_4$: 8% Er nanocrystals were stored in 3 mL cyclohexane. The nanoparticles have diameter of ~100 nm and height of 70 nm.

$NaYbF_4$: 10% Gd, 8% Er@$NaYbF_4$: 8% Er@$NaYbF_4$: 8% Er: the nanoparticles were synthesized by a similar procedure used for $NaYbF_4$: 10% Gd, 8% Er@$NaYbF_4$: 8% Er, except that $NaYbF_4$: 10% Gd, 8% Er@$NaYbF_4$: 8% Er was used as the precursor. The resulting hexagonal prisms have diameter of ~130 nm and height of 80 nm.

Finally, the inactive $NaYF_4$ layer was added using a similar procedure used for synthesizing $NaYbF_4$: 10% Gd, 8% Er@$NaYbF_4$: 8% Er, with 0.40 mmol $YCl_3 \cdot 6H_2O$ replacing 0.40 mmol $RECl_3 \cdot 6H_2O$ (92% Yb^{3+}, 8% Er^{3+}), and $NaYbF_4$: 10% Gd, 8% Er@$NaYbF_4$: 8% Er replacing $NaYbF_4$: 10% Gd, 8% Er. The purified $NaYbF_4$: 10% Gd, 8% Er@$NaYbF_4$: 8% Er@$NaYbF_4$: 8% Er@$NaYF_4$ was stored in 3 mL cyclohexane. The hexagonal prism UCNPs have diameter of ~160 nm diameter and height of 90 nm.

Supplementary Information Figures

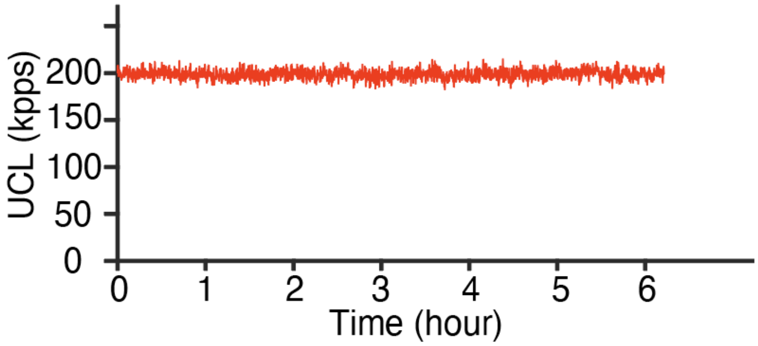

Supplementary Fig. 1. Long-term stability of upconversion luminescence of single CSS nanoparticles, $NaYF_4$@ $NaYbF_4$: 8 % Er@$NaYF_4$ with 27 kW/cm^2 of 978 nm excitation. For intensities up to 6 $MWcm^{-2}$, we observed no evidence of photobleaching.

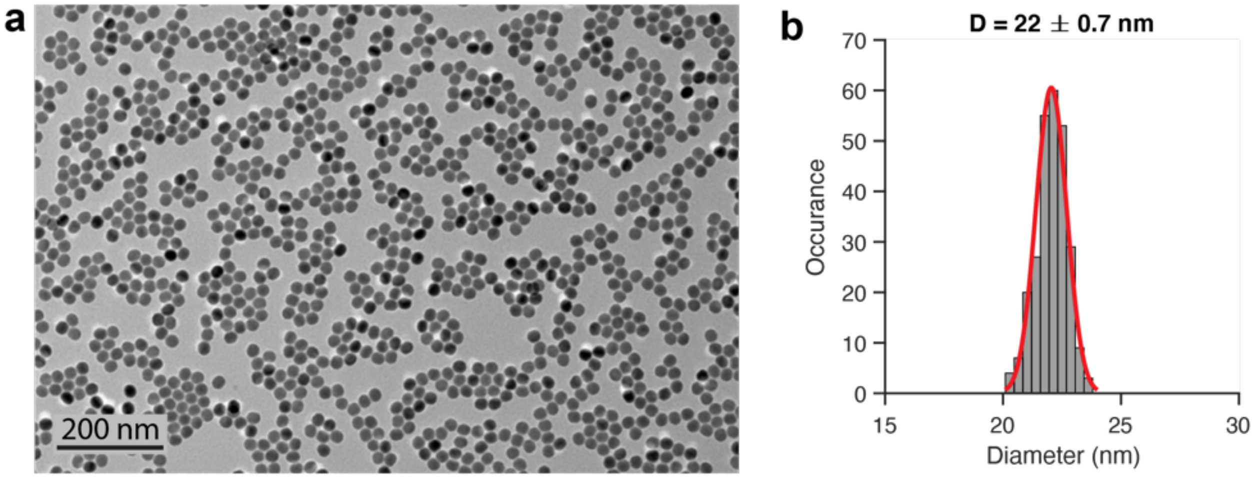

Supplementary Fig. 2. TEM image (a) and size distribution (mean ± s.d) (b) of the core-only $NaYF_4$: 20%Yb, 2% Er. Synthesis and detailed characterizations of this UCNP has been reported in our previous publication[12]. n = 3 independent experiments.

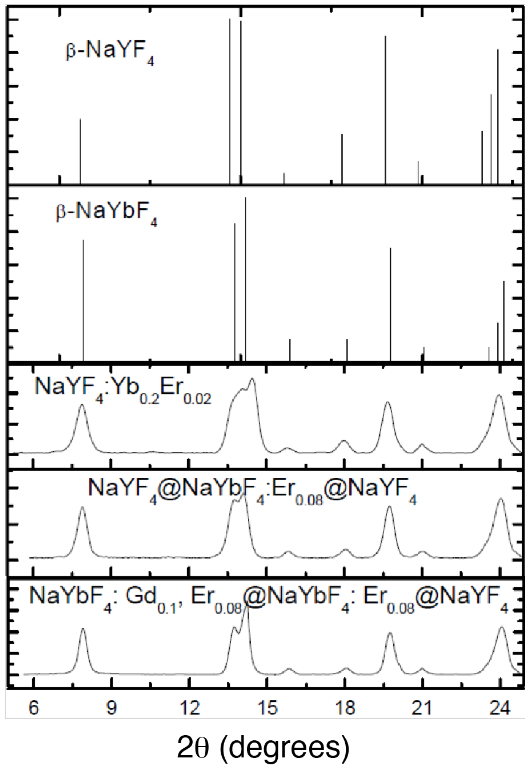

Supplementary Fig. 3. XRD patterns of the UCNPs and standard β-phase $NaYF_4$ and $NaYbF_4$.

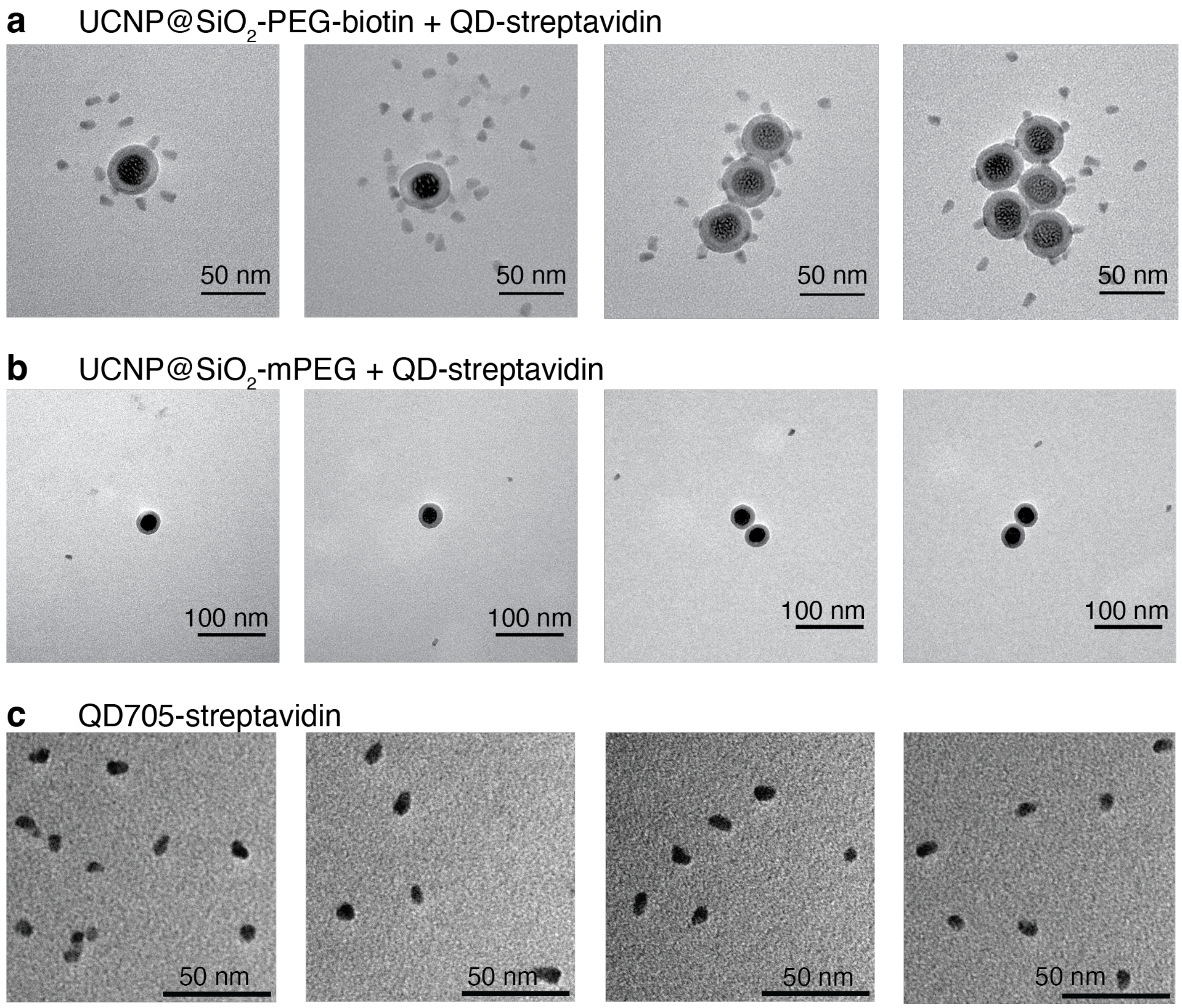

Supplementary Fig. 4. TEM images of mixture of streptavidin coated quantum dots with (a) $NaYF_4$: 20% Yb, 2 % Er@SiO_2-PEG-biotin and (b) $NaYF_4$: 20% Yb, 2 % Er@SiO_2-mPEG. Larger field of views were displayed in (b) in order to show the presence of unbound QD-streptavidin, demonstrating that the bound QDs in (a) are due to specific biotin-streptavidin interactions rather than non-specific sticking. (c) TEM images of QD705-streptavidin alone. n = 3 independent experiments.

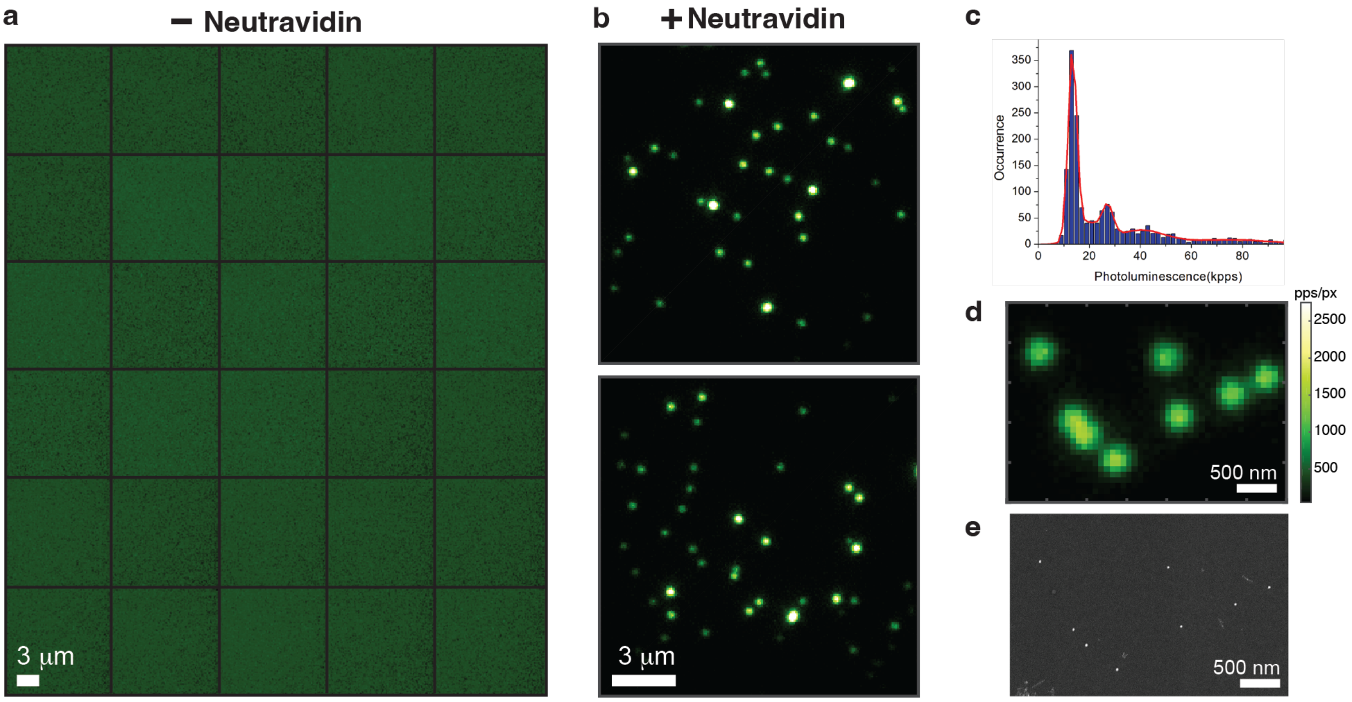

Supplementary Fig. 5. Wide-field fluorescent images of $NaYF_4$: 20% Yb, 2 % Er@SiO_2-PEG-biotin on biotinylated PEG coverslips in the absence (a) and presence (b) of neutravidin. When btn-UCNPs were incubated on coverslips for 5 minutes without neutravidin and subsequently washed away, no particles were detected in at least thirty field of views. Under background-free imaging condition (976 nm excitation) and the absence of UCNPs, it was necessary to check the focus was maintained during image acquisition. For every five images taken, we switched to 532 nm excitation and used the autofluorescence from the coverslip surface to re-adjust the focus. The fact that no UCNPs were detected in 30 FOVs while on average 65 UCNPs were found in the presence of NeutrAvidin allowed us to establish at least 2000:1 specific versus non-specific binding of btn-UCNPs. n = 10 independent experiments. (c) Histogram of single-particle brightness from the experiment shown in panel (b). The histogram shows a major peak at $13.3 \pm 1.6\ kpps$ (mean ± s.d) which is similar to the single particle brightness of the precursor UCNP ($NaYF_4$: 20% Yb, 2 % Er) at $15.5 \pm 1.6\ kpps$. The histogram also shows peaks at ~26 $kpps$ and ~40 $kpps$, corresponding to dimers and trimers. (d) Optical image of $NaYF_4$: 20% Yb, 2 % Er UCNPs. (e) SEM image of the same field of view shown in panel (d), demonstrating that the individual PSFs in our optical images correspond to single UCNPs. n = 3 independent experiments.

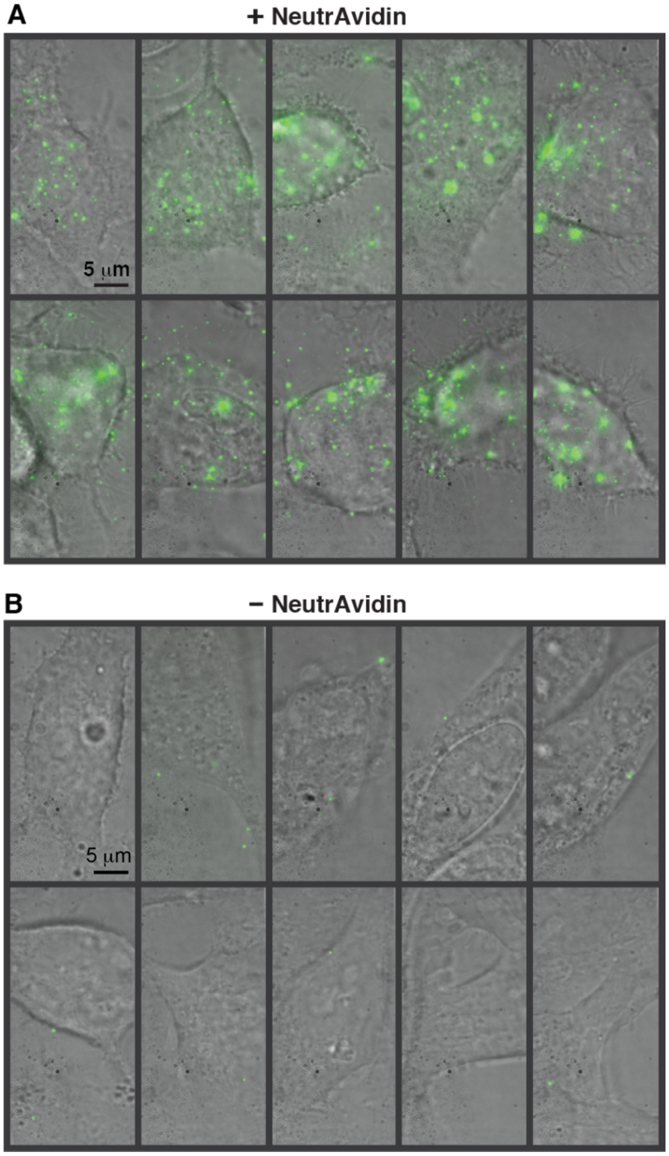

Supplementary Fig. 6. Other examples of live-cell imaging of btn-UCNPs on HeLa cells whose membrane proteins have been biotinylated. Overlay of brightfield images of HeLa cells and maximum-intensity-projected luminescence images of biotinylated-UCNPs (btn-UCNPs) in the presence (a) and absence (b) of NeutrAvidin. n = 20 cells.

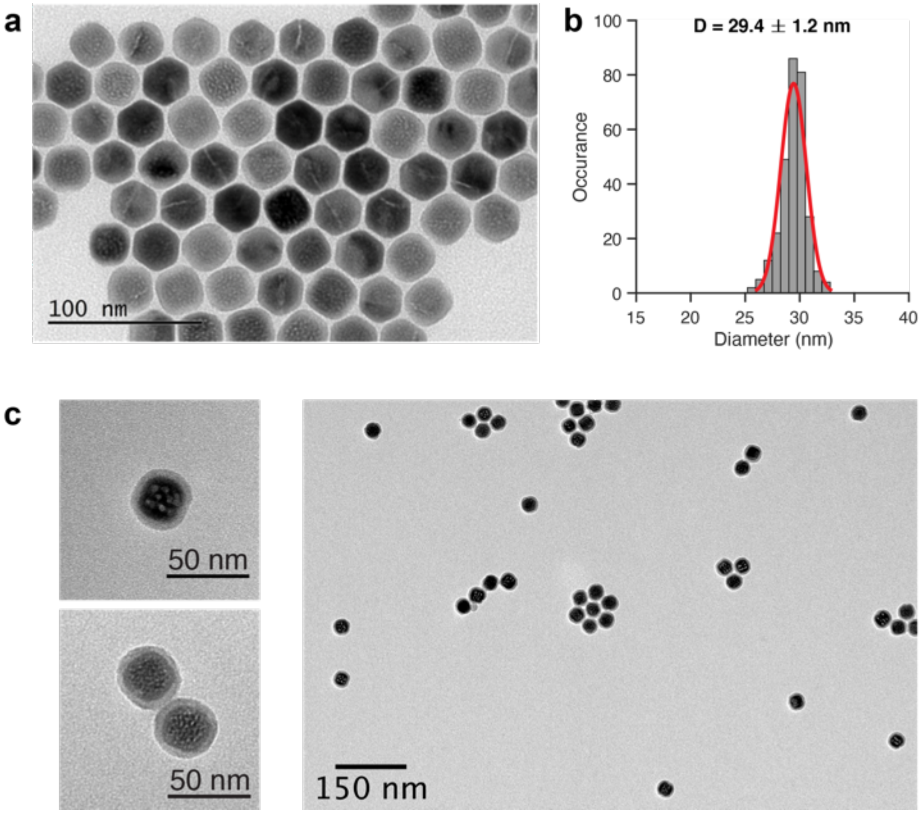

Supplementary Fig. 7. TEM image (a) and size distribution (mean ± s.d) (b) of the core-shell-shell $NaYF_4$@ $NaYbF_4$:8% Er@$NaYF_4$. Synthesis and detailed characterizations of this UCNP has been reported in our previous publication[12]. (c) TEM images of silica coated UCNPs. The silica shell thickness is ~ 4 nm. n = 3 independent experiments.

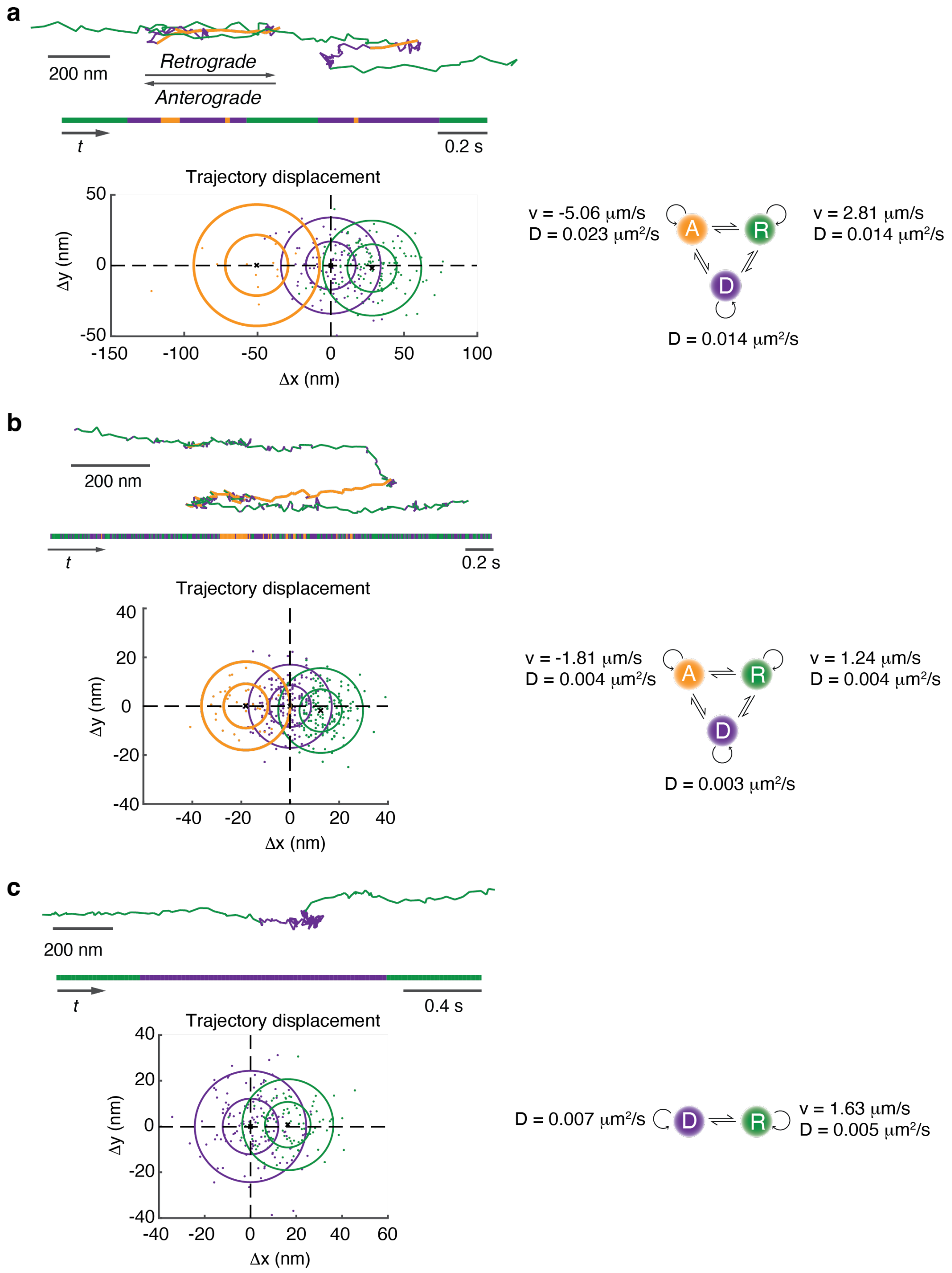

Supplementary Fig. 8. HMM-Bayes analysis of the retrograde transport in rat DRG (A) and human iNs (B), corresponding to the trajectories shown in Fig. 2B and 2C. For each trajectory, scatterplots of the displacements are shown on the bottom and each point is colored purple, green, and orange indicating the inferred diffusive, retrograde (distal axon to soma) or anterograde (soma to distal axon) transport, respectively. Circles indicate one and two standard deviations of the displacements within each state. The inferred diffusion coefficients and velocities are shown on the right. (C) One example of iN trajectory that shows only retrograde and diffusion states.

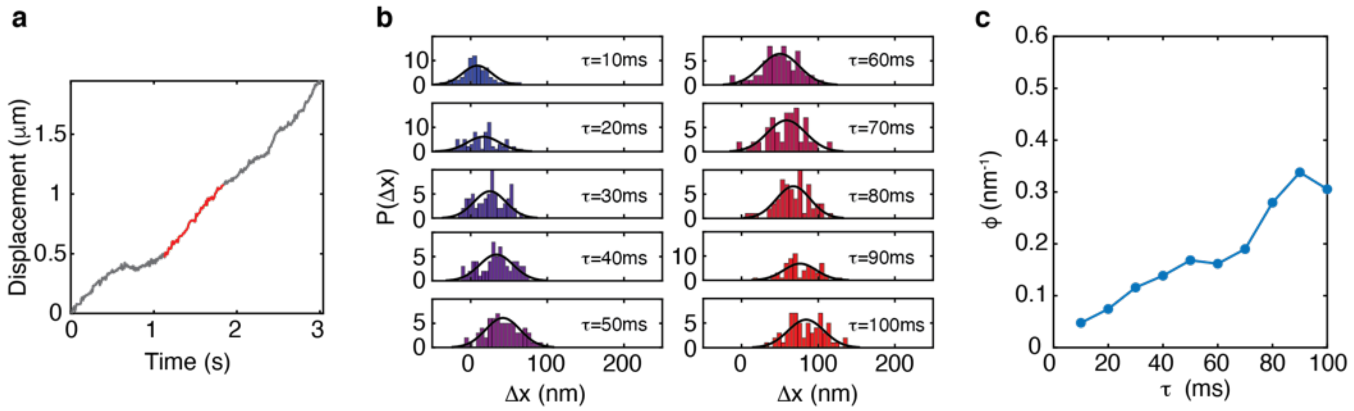

Supplementary Fig. 9. One example of constant velocity segment where ϕ does not reach a steady-state asymptotic value. (a) Displacement curve of retrograde transport in rat DRG neurons. (b) Probability distribution of displacement Δx at different time delays (10 ms to 100 ms). (c) Relaxation curve of the effective entropy ϕ, calculated from the mean and variance of the Gaussian distributed $P(\Delta x)$.

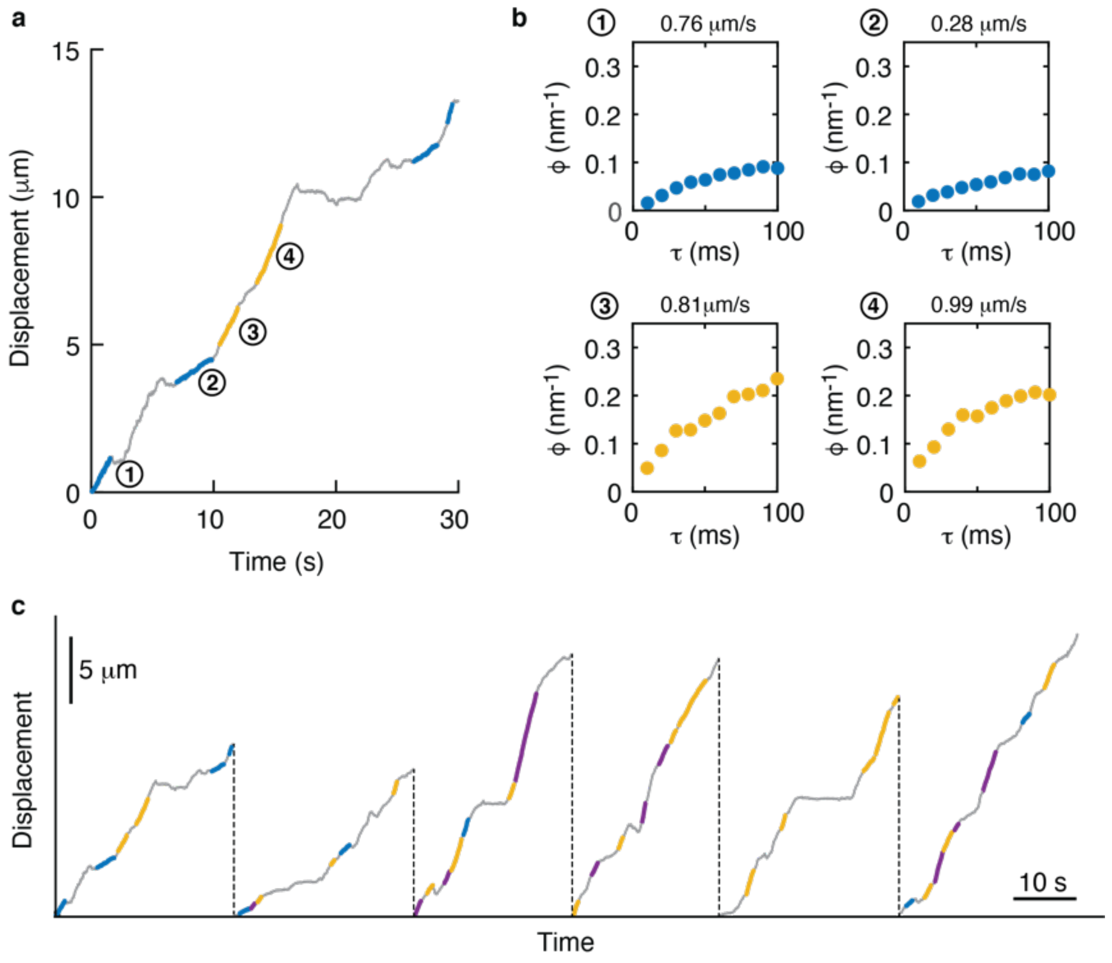

Supplementary Fig. 10. Example showing displacement segments where the motion approaches different asymptotic values of $\phi \equiv 2\mu/s^2$ during the retrograde transport. (a) The displacement curve of retrograde transport in rat DRG neurons. Colored lines mark the constant velocity segments that show clear relaxation of ϕ curves. (b) Relaxation curves of the effective entropy calculated for each of the four constant velocity segments marked in (a). The average velocities of these segments are shown on the top of each panel. (c) More displacement curves from the same cargo. Color codes follow Fig. 3f where blue, yellow, and purple indicate one, two, and three dynein motors, respectively.

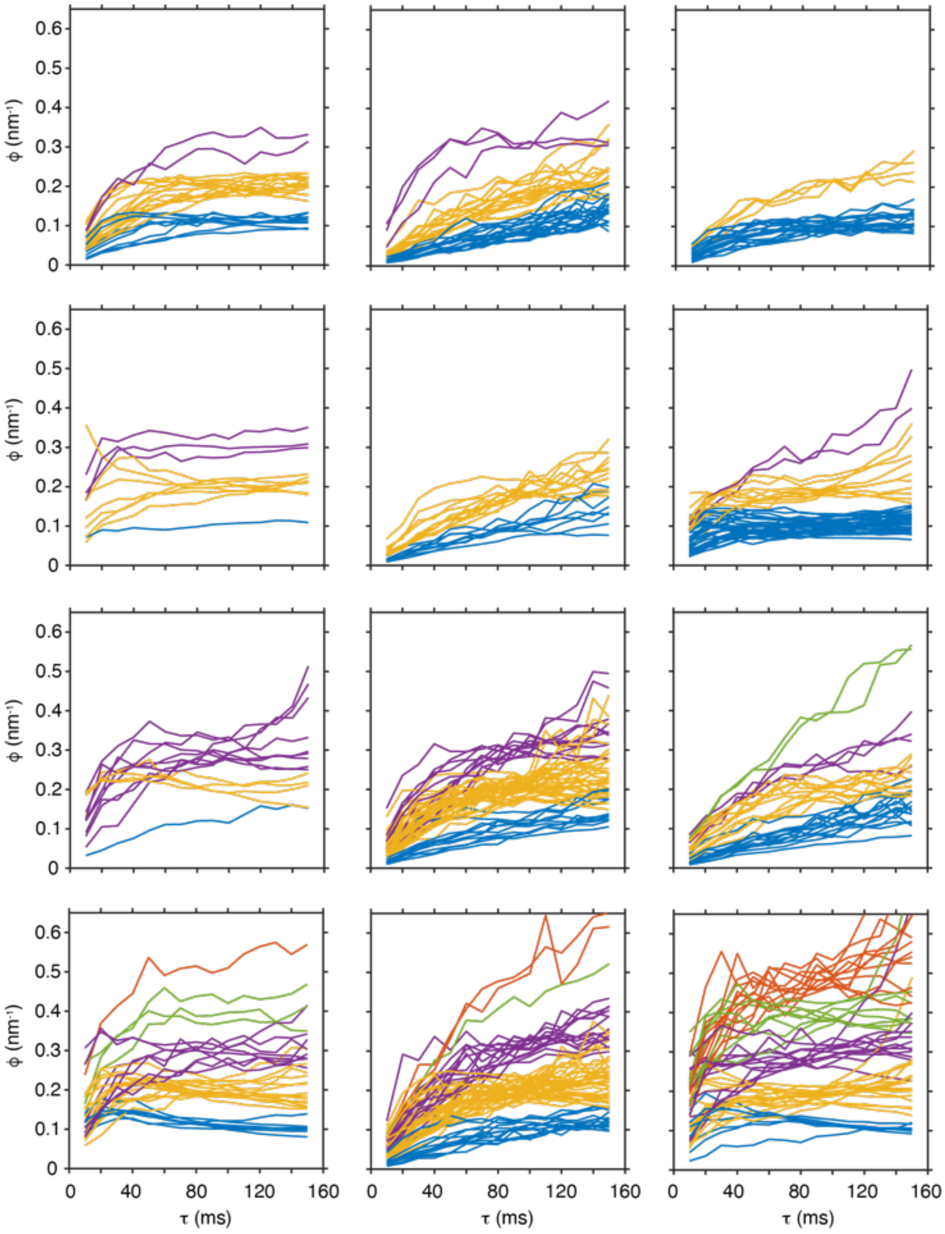

Supplementary Fig. 11. Effective entropy ϕ curves for twelve individual retrograde endosomes in DRG neurons. In addition to the example shown in the main text which exhibits quantized ϕ values of 0.1, 0.2, and 0.3, some endosomes only show ϕ values of 0.1 and 0.2, while some endosomes have ϕ values up to 0.5. The number of ϕ curves for each endosome depends on the length of the recorded trajectories. We note that a fully asymptotic (steady-state) value of ϕ is rarely achieved during the constant velocity segments, such as shown in Supplementary Fig. 12.

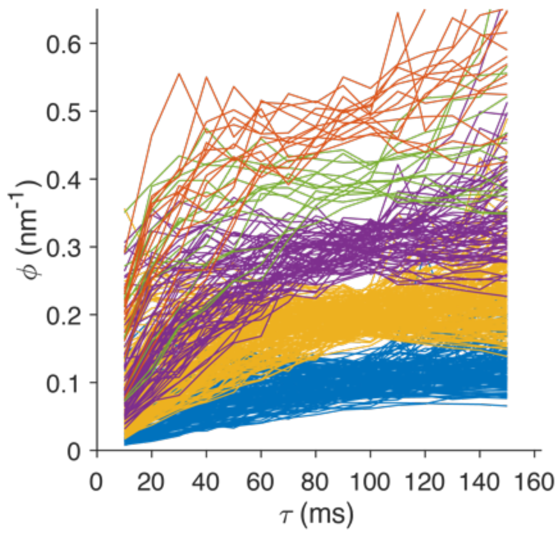

Supplementary Fig. 12. Pooled effective entropy ϕ curves from 12 different retrograde cargoes in rat DRG neurons.

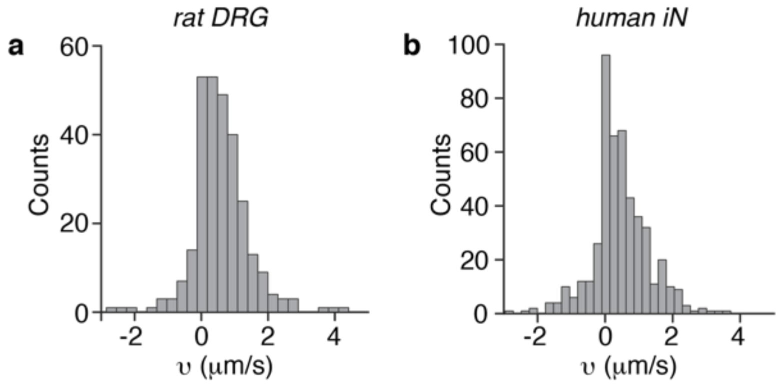

Supplementary Fig. 13. Velocity distribution of single retrogradely transported cargo in (a) rat DRG and (b) human induced neurons. No clear quantized peaks are observed in these velocity distributions.

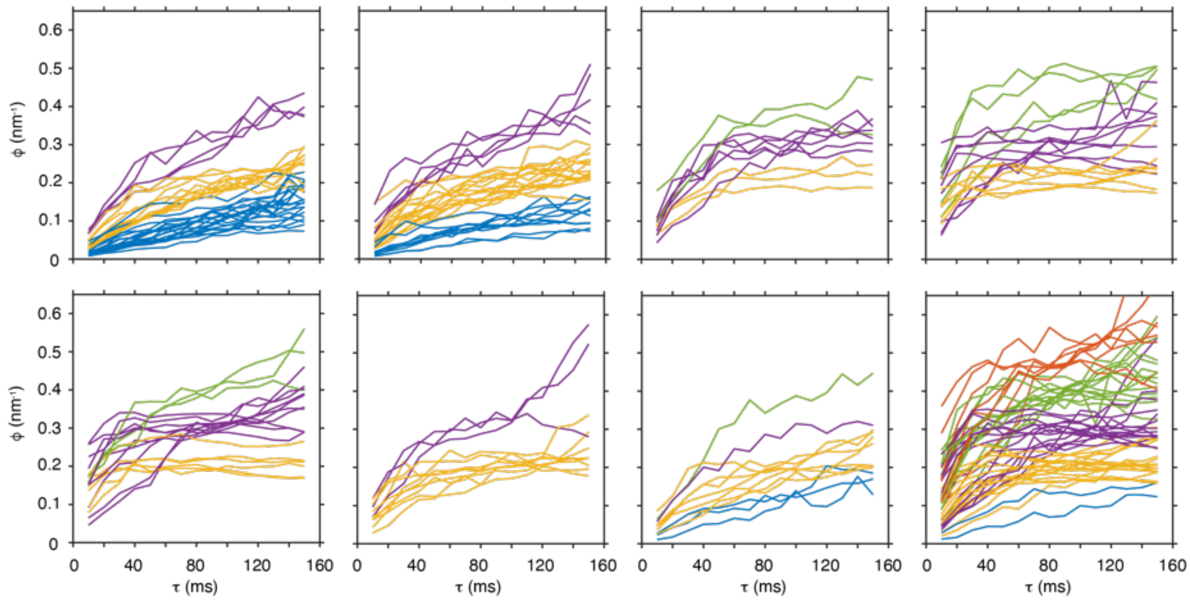

Supplementary Fig. 14. Effective entropy ϕ curves for eight individual retrograde endosomes in human induced neurons.

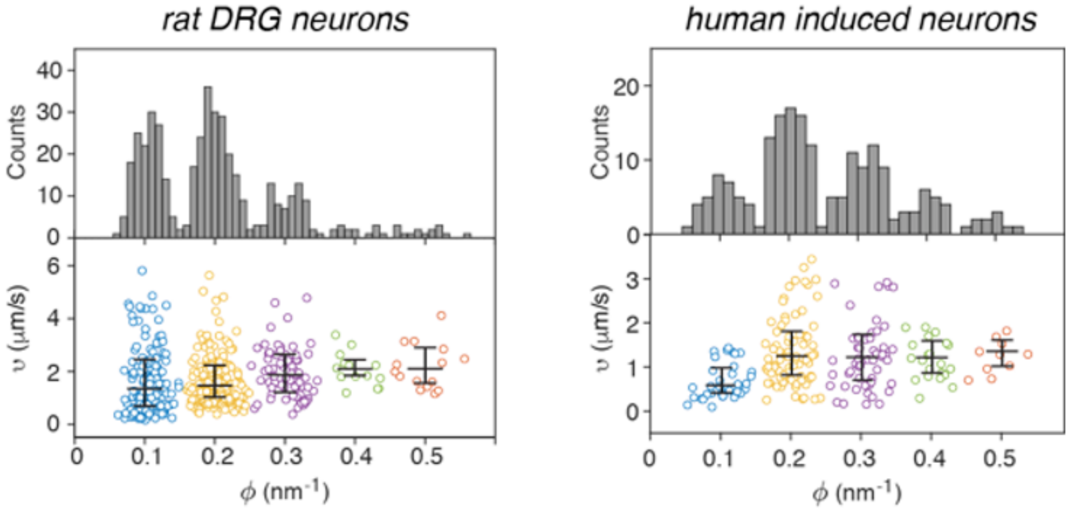

Supplementary Fig. 15. Fluctuation theorem analysis results for rat DRG neurons and human induced neurons. The ϕ was averaged from τ = 80 ms to 120 ms. n = 430 and 194 for rat DRG and human iN, respectively. The average (± s.e.m.) number of dynein motors were 2.01 ± 0.05 and 2.50 ± 0.08 for rat DRG and human iNs, respectively.

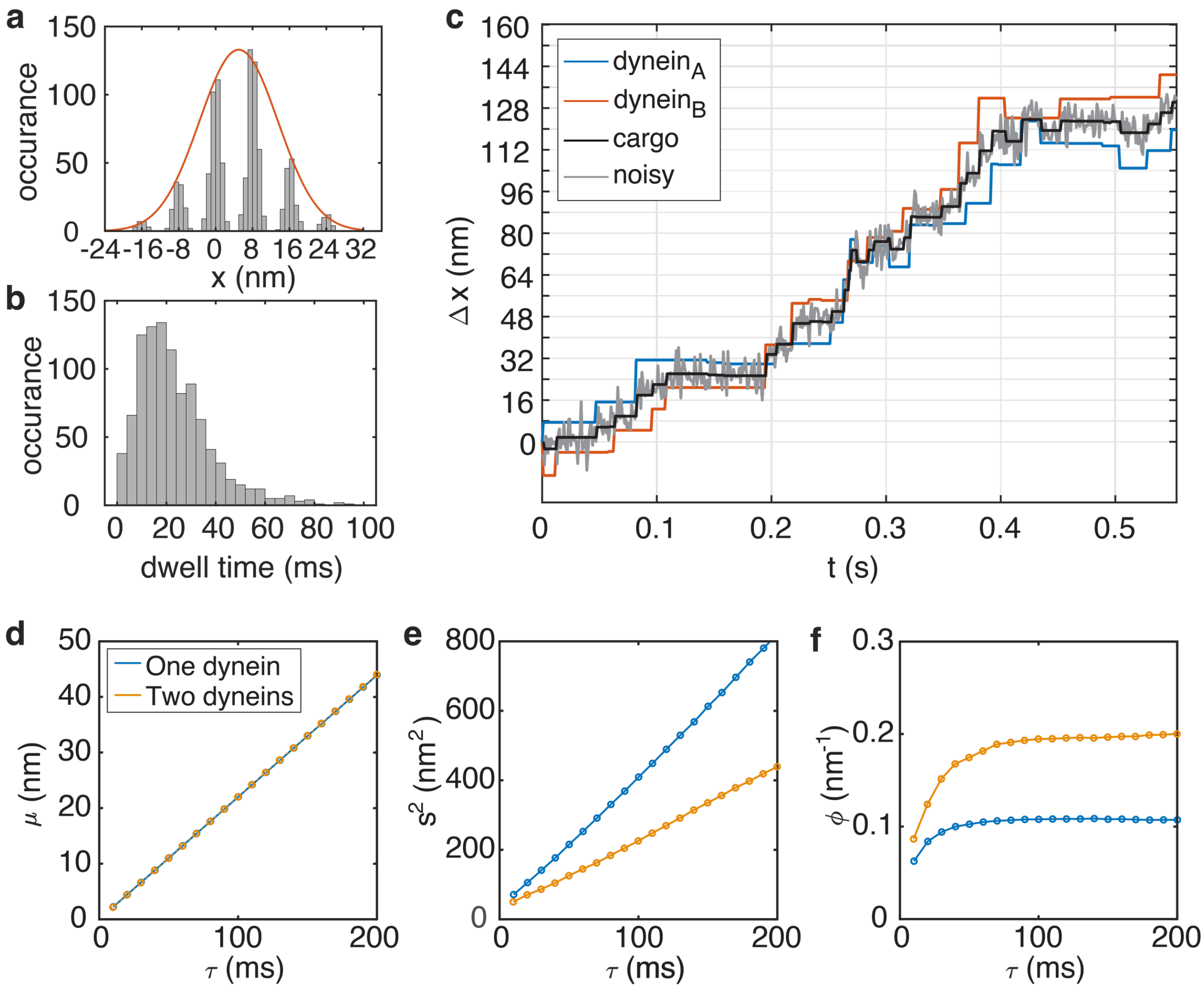

Supplementary Fig. 16. (a) The simulation input of the step-size histogram for one dynein dimer motor, which was generated based on the experimentally measured cargo step-size histogram at $22°C$ shown in Supplementary Fig. 21. One thousand steps were simulated. Multiple Gaussian distributions with a $\sigma = 1\ nm$ were placed at every $8\ nm$ to represent the allowed binding sites on the MT. The center of the overall distribution (red curve) is at $5\ nm$. (b) The simulation input of the dwell time distribution based on the experimentally measured distribution at $37°C$ shown in Fig. 4c. (c) Simulated stepping trajectory for two dynein dimers (dynein$_A$ and dynein$_B$). To simulate a trajectory for a cargo with only one dynein dimer, the trajctory of dynein$_A$ was used. To simulate a trajectory for a cargo with two dynein dimers, the average position of dynein$_A$ and dynein$_B$ was taken (black curve). Finally, to mimic the experimental noise, noise of $\sigma_{noise} = 4\ nm$ was added (grey line). (d) The mean and (e) variance of the probability distributions $P(\Delta x(\tau))$ as a function of the time delay, for the one-dynein system (blue line) and two-dynein system (orange line). The analysis of the simulated trajectory was performed the same way as for the experimental data, as illustrated in Fig. 3b-3d. The simulations showed that the mean displacement did not change (the two curves overlap significantly) while the variance dropped by a factor of 2 from the one-dynein to two-dynein system. (f) The corresponding ϕ as a function of the time delay. The ϕ value for the two-dynein system is twice of that of the one-dynein system, consistent with the anaylitical treatment given in the Main Text.

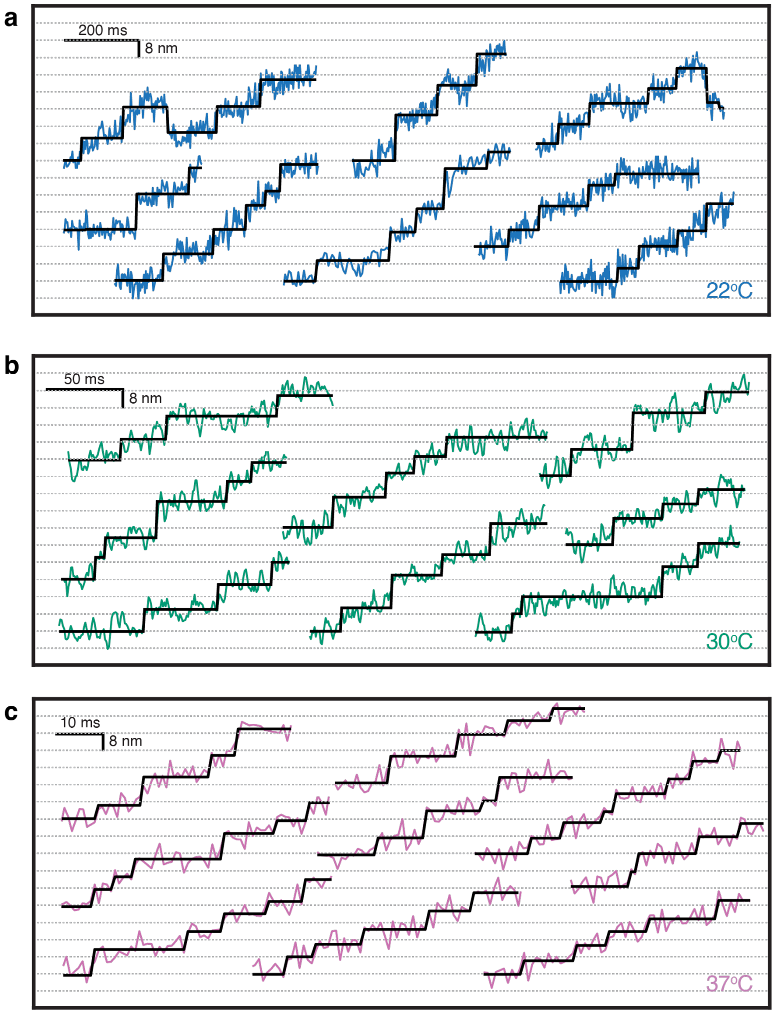

Supplementary Fig. 17. More retrograde transport step-size traces as shown in Fig. 4. Displacement traces are broken into segments and displaced vertically for better visualization. The blue, green, and red lines are the raw trajectories at (a) 22 $°C$, (b) 30 $°C$, and (c) 37 $°C$, respectively. The black lines mark the steps detected by step-finding algorithm. Dynein steps exhibit 8 nm and larger steps. Notice that the different time scales are used at different temperatures.

A priori, it may be possible that the 80 × 60 nm core-shell-shell UCNPs used at 22°C and 30 °C and the larger 160 × 90 nm particles used at 37 $°C$ could affect the average speed of transport. In comparing the speed distribution of this work with our earlier work using 10 nm quantum dots[13], the velocity distributions were similar[13]. In the work of Hayashi, et al.[14], lipid dyes were used to stain the entire neuronal endosome. The fluorescence intensity is proportional to the surface area of the endosome, and hence the square root of the fluorescence intensity scales with the radius (the "size") of the endosomes. In Fig. 6b of Hayashi et al.[14], if we use our interpretation of χ^*, which is equivalent to our ϕ, as the number of motors, the distribution of the number of motors increases only very slightly with vesicle size. In their Fig. 6a, the velocity distribution also increases slightly with the number of motors.

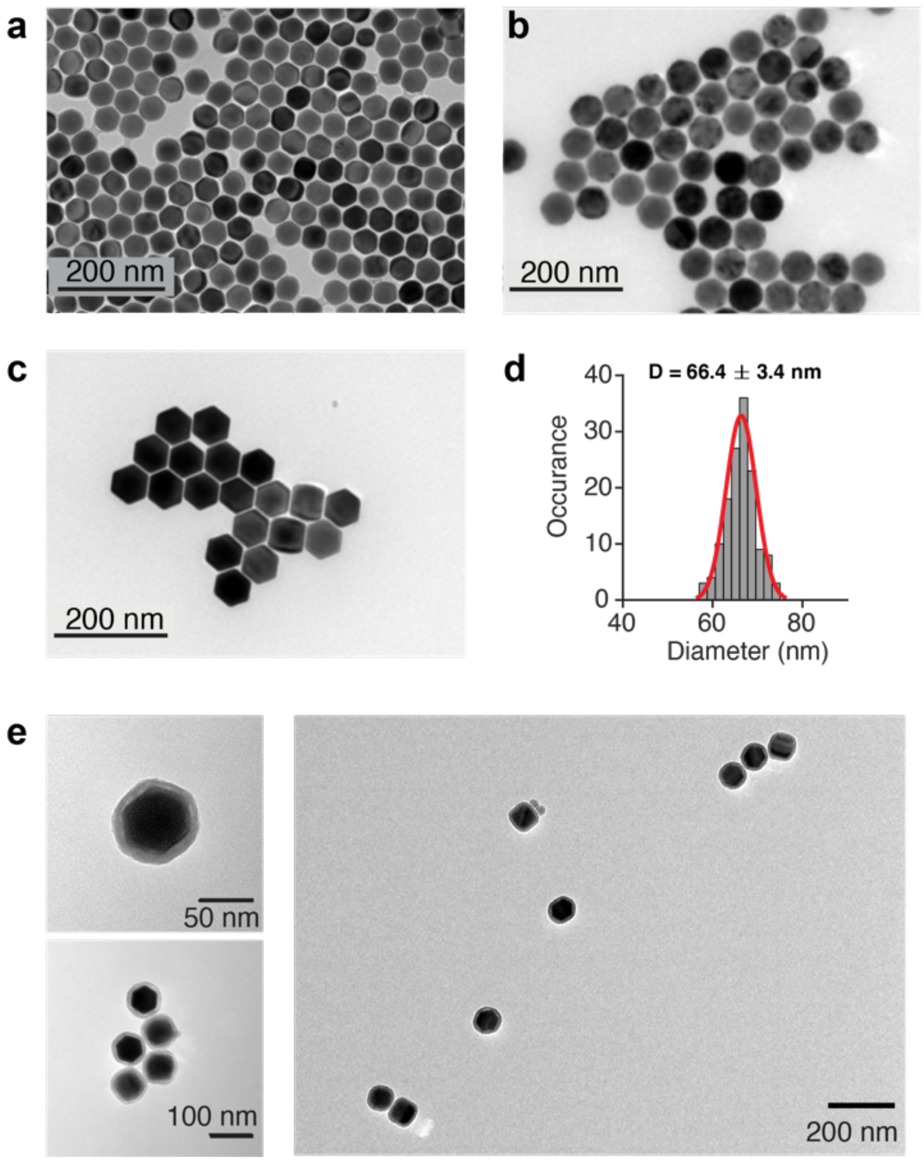

Supplementary Fig. 18. TEM images of (a) $NaYbF_4$: 10%Gd, 8% Er, (b) $NaYbF_4$: 10% Gd, 8% Er@$NaYbF_4$: 8% Er, and (c) $NaYbF_4$: 10% Gd, 8% Er@$NaYbF_4$: 8% Er@ $NaYF_4$. (d) Size distribution (mean ± s.d) of $NaYbF_4$: 10% Gd, 8% Er@$NaYbF_4$: 8% Er@ $NaYF_4$. (e) TEM images of silica coated $NaYbF_4$: 10% Gd, 8% Er@$NaYbF_4$: 8% Er@ $NaYF_4$. The silica shell thickness is ~8 nm. n = 3 independent experiments.

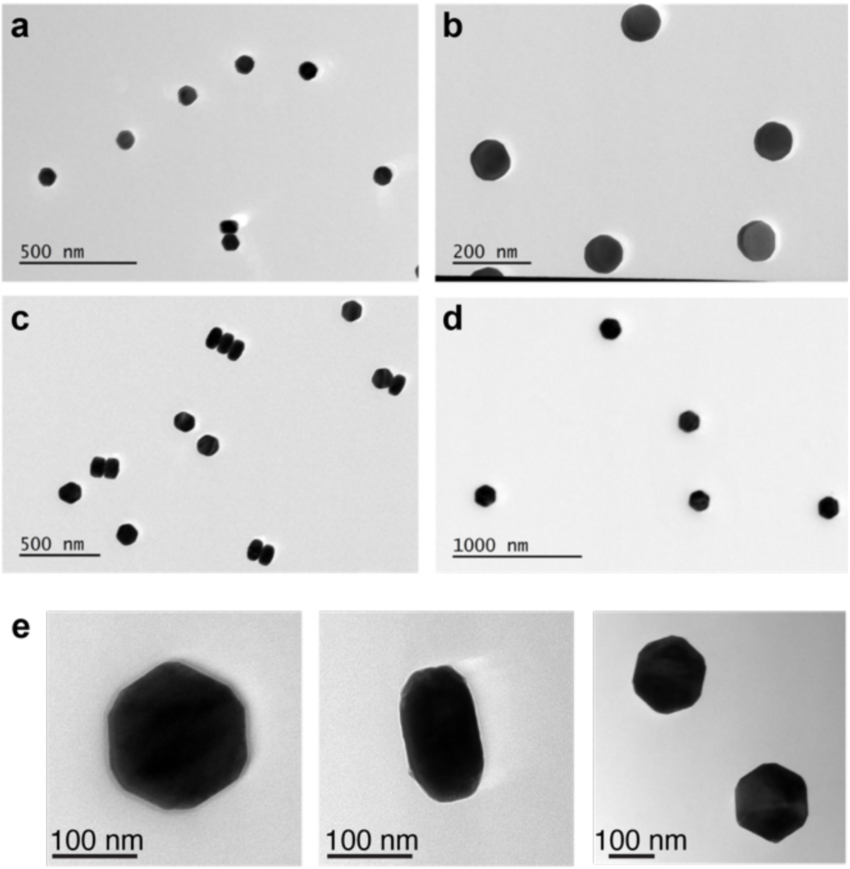

Supplementary Fig. 19. TEM images of (a) $NaYbF_4$: 10% Gd, 8% Er (diameter of ~80 nm and height of ~60 nm), (b) $NaYbF_4$: 10% Gd, 8% Er@$NaYbF_4$: 8% Er (diameter of ~100 nm and height of ~70 nm), (c) $NaYbF_4$: 10% Gd, 8% Er@$NaYbF_4$: 8% Er@$NaYbF_4$: 8% Er (diameter of ~130 nm and height of ~80 nm), and (d) $NaYbF_4$: 10% Gd, 8% Er@$NaYbF_4$: 8% Er@$NaYbF_4$: 8% Er@$NaYF_4$ (diameter of ~160 nm and height of ~90 nm). (e) TEM images of silica coated $NaYbF_4$: 10% Gd, 8% Er@$NaYbF_4$: 8% Er@$NaYbF_4$: 8% Er@$NaYF_4$. The silica shell thickness is ~4 nm. n = 3 independent experiments.

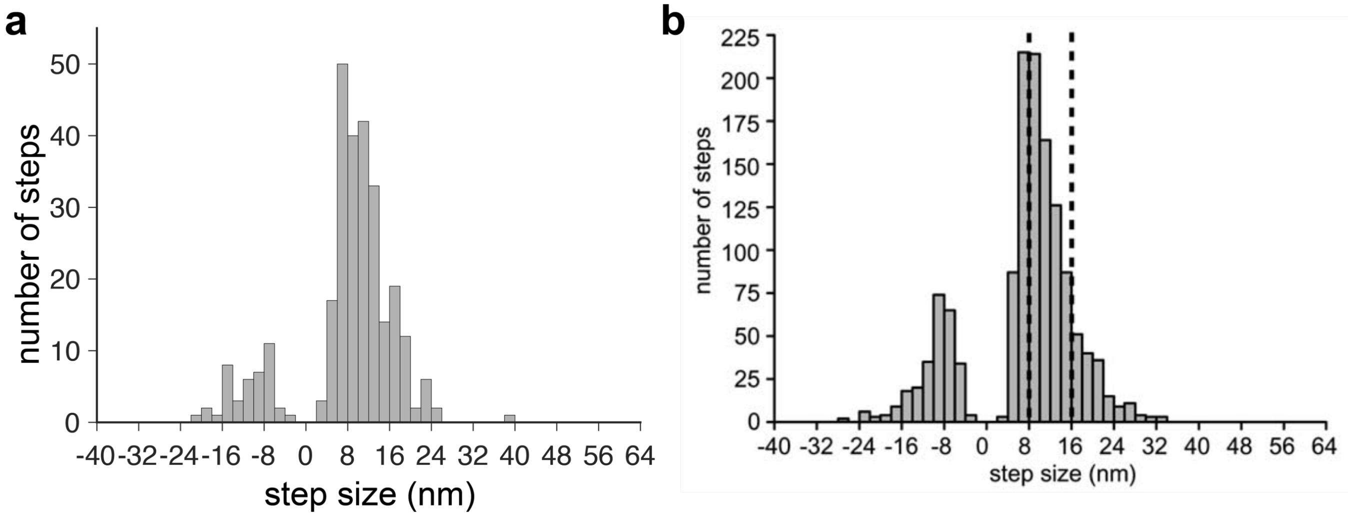

Supplementary Fig. 20. (a) The step-size histogram of dynein in live neurons at 22 °C displayed multimodal distribution with a major peak at 8.3 nm and larger (12-24 nm). (b) The step-size histogram of purified dynein measured in *in vitro* experiments at 22 °C. Adapted from Ref. [6] with permission.

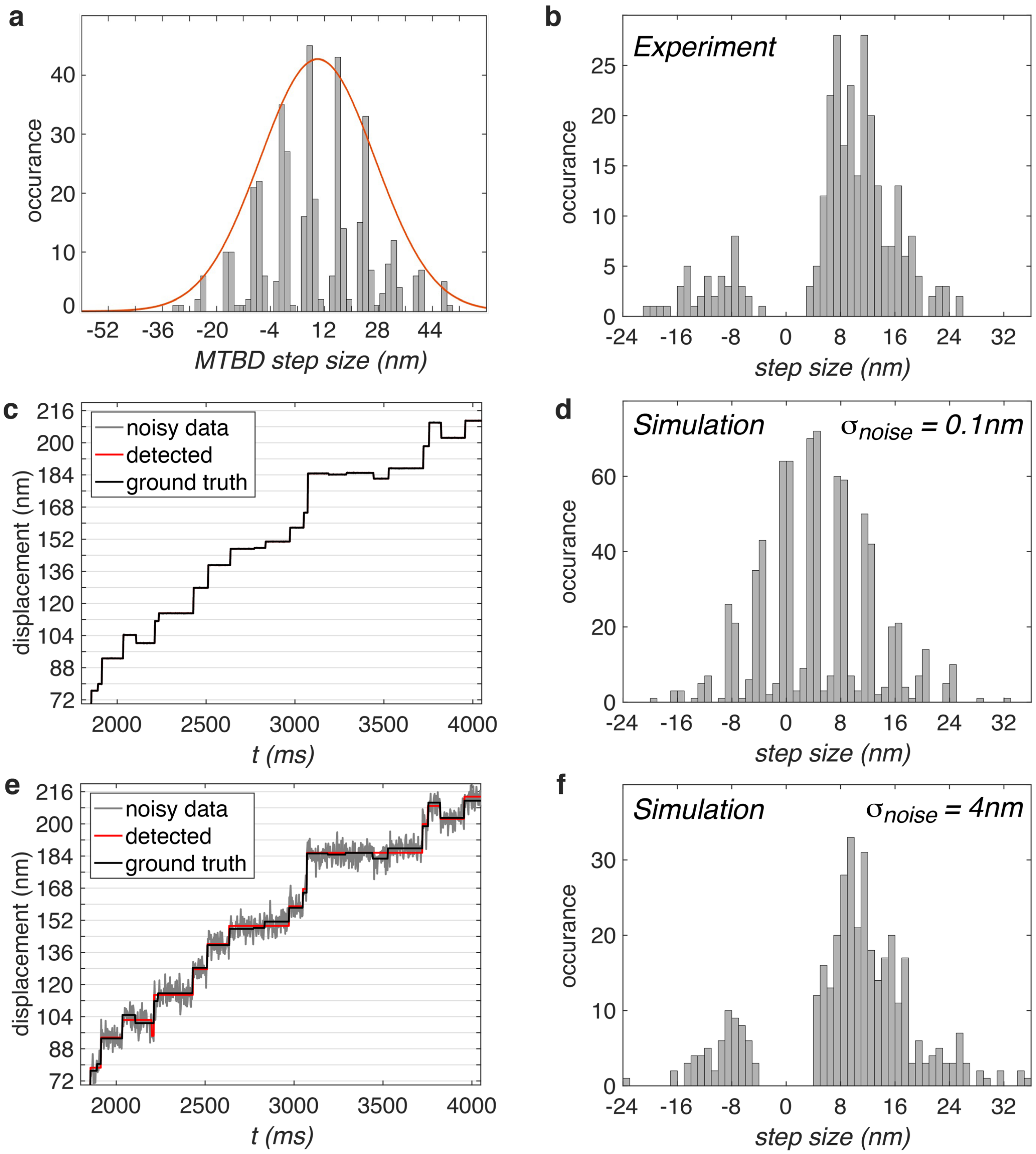

Supplementary Fig. 21. Simulation of the step-resolved trajectory of a single dynein motor and validation of our step-finding algorithm. (a) The simulation input of the MTBD step-size histogram, which was generated based on the experimentally measured cargo step-size histogram at $22°C$ shown in panel (b). We assume that if one motor domain of the dynein motor takes a step while the other has not stepped, the motion of the vesicle will be half the distance of the stepping motor domain. For this reason, the width of the probability distribution of a dynein motor domain in Fig. 21a was taken to be twice of that of the cargo distribution. We further assume that cargo steps smaller than $\sim 4\ nm$ were not detected in our experiment. For a dynein motor consisting of two motor domains, if one of motor domains moves by $\sim 8\ nm$, and the other does not move, the vesicle will move by $4\ nm$. Since the

technical noise is $\sim 4\ nm$, our step-finding algorithm may not detect these small steps reliably. However, the steps still add to the overall displacement of the vesicle.

To construct the "ground truth" step size distribution for a MTBD, multiple Gaussian distributions with a $\sigma = 1\ nm$ were placed at every $8\ nm$ to represent the allowed binding sites on the MT. The center of the overall distribution (red curve) is at $10\ nm$. (c) Simulated trajectory of the step movement of the cargo based on two independent dynein motor domains. Noise of $\sigma_{noise} = 0.1\ nm$ was added. (d) The detected step-size histogram from the trajectory in Supplementary Fig. 21c. In a study of live cell tracking of dynein with very high temporal and spatial resolution, $4\ nm$ step size intervals for step sizes above 8 nm were clearly resolved, as shown in Fig. 4c of Ref. [8]. The authors chose not to display the distribution of backward steps. However, in Figs. 8B, 8C, of their Supporting Information[8], $\pm 4\ nm\ and - 8\ nm$ steps were clearly detected, thus confirming our simulation of Supplementary Fig. 21d. (e) When we simulated displacement trajectories with added noise $\sigma_{noise} = 4\ nm$, the step-finding algorithm produces a step size distribution (f) that is very similar to the detected step-size histogram in Supplementary Fig. 21b.

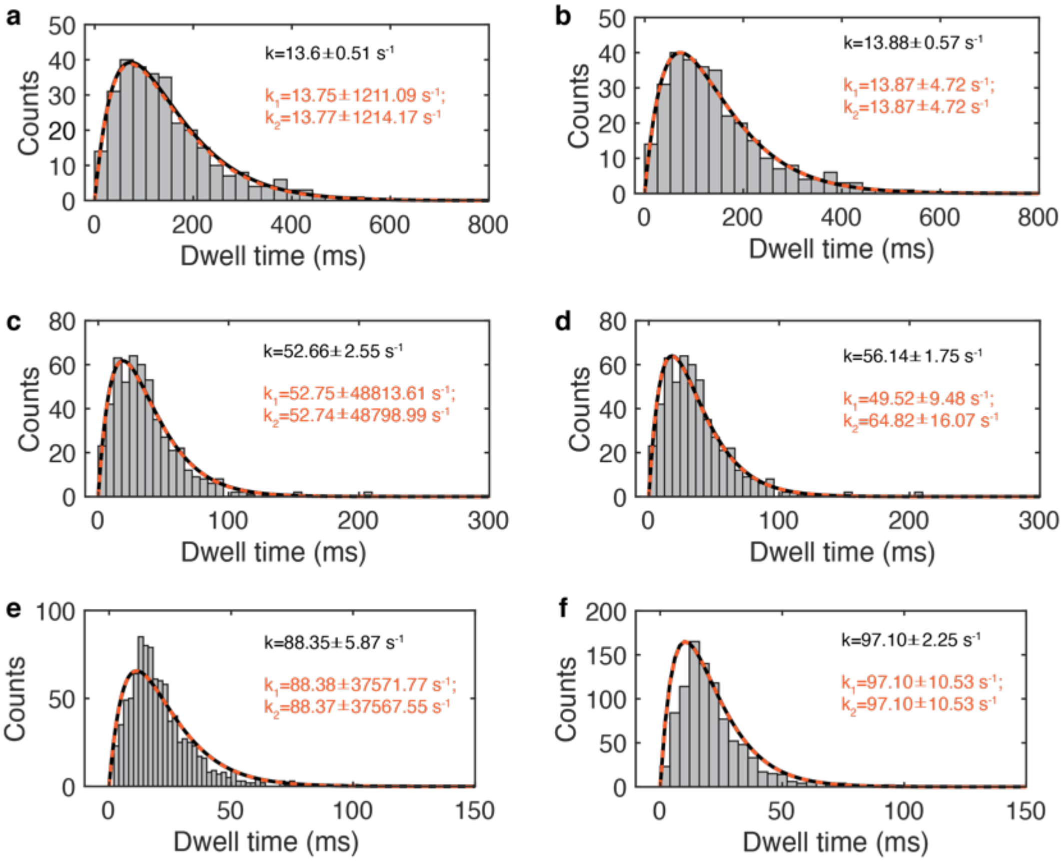

Supplementary Fig. 22. Dwell-time histograms for retrograde transport in live rat DRG neurons at 22 $°C$ (a, b), 30 $°C$ (c, d), and 37 $°C$ (e, f). The dwell-time histograms at all three temperatures fit well to a kinetic scheme consisting of stepping cycle described two independent but sequential rate constants k_1 and k_2. In this model, the dwell time distribution is $P(t) = \int_0^t P_{k_1}(\tau)P_{k_2}(t-\tau)d\tau$. If $k_1 = k_2$, the fits are given by $P(t) = \tau k^2 e^{-k\tau}$ (black curves). For $k_1 \neq k_2$, $P(t) = \frac{k_1 k_2}{k_1 - k_2}(e^{-k_2\tau} - e^{-k_1\tau})$, the fits are the orange dash lines.

Fitting was done using two different methods: (1) fit to binned histogram using *lsqnonlin* function in Matlab (a, c, e); and (2) directly fit the raw dwell times using Maximum Likelihood Estimates (MLE) (b, d, f). Note that the fitting results using (1) can vary with the choice of bin size, whereas the fitting results using (2) does not depend on the bin size because it directly fits to the raw dwell time data points. We stress here that fitting the dwell times to a pre-binned histogram is subject to bias, and the bins could be chosen to suppress the rapidly rising portion of the dwell time distributions.

The error bars reported are 95% confidence interval for (1) and standard error for (2), which is the square root of the covariance matrix from MLE. In all cases except for (d), the fitting results indicated a model of the same rates. Even in (d), the single rate constant ($k = 56.14 \pm 1.75\ s^{-1}$) is within the error bars of the two different rates ($k_1 = 49.52 \pm 9.48\ s^{-1}; k_2 = 64.82 \pm 16.07\ s^{-1}$). Therefore, we constrained the model to having the same rate, $P(\tau) = \tau k^2 e^{-k\tau}$, which resulted in smaller error bars.

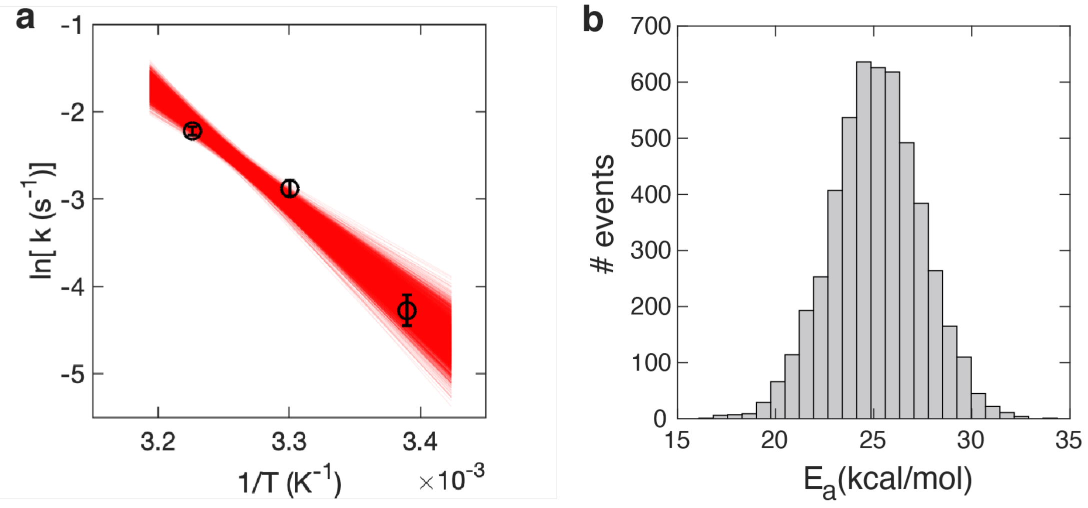

Supplementary Fig. 23. (a) Arrhenius plot of the equal rate constants $k \equiv k_1 = k_2$ (mean ± s.e) at $22\ °C$, $30\ °C$, and $37\ °C$. The uncertainties in the k's was propagated to obtain the uncertainties of $\ln(k)$ and shown as the error bars. We randomly generated a new $\ln(k)$ at each temperature based on the uncertainties of $\ln(k)$, and then fit a linear line (red line). The process was repeated for 5000 times to obtain the uncertainty in the fitted slope. (b) Histogram of the activation energy from 5000 fittings. Activation energy of $25.1 \pm 2.3\ kcal/mol$ (mean ± s.e) was obtained. The derived activation energy is only logarithmically dependent on the measured rate constants obtained by tracking different vesicles.

Supplementary Fig. 20 shows the comparison of our step size distribution to the one reported in Reck-Peterson *et al.* [6]. Since the distributions are very similar, we conclude that the dwell time distribution in Fig. 4c at 22 ℃ of the Main Text is is due to a single dynein motor. The fact that the three dwell time distributions at different temperatures are consistent with a single activation energy allows us to infer that the dwell time distributions taken at the higher temperatures with different dynein motor systems must have the same number of motors. To see this, suppose the dwell-time distribution taken at higher temperatures were due to two motors. If this were true, the stepping rates would be twice as fast at the same temperature and the stepping rate could not be described as due to two thermally activated rates. Thus, we conclude that all the single step data taken at different temperatures and with different vesicles are all measurements of different single motor systems.

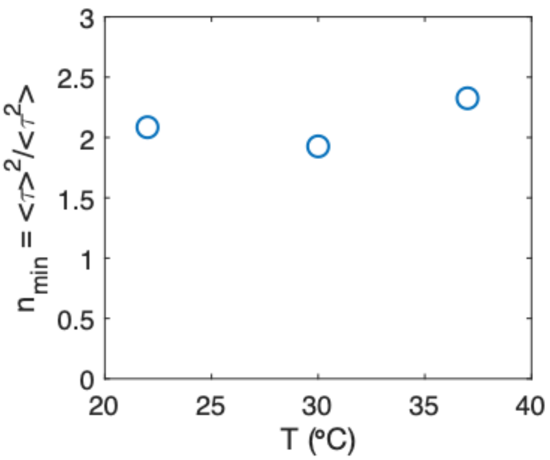

Supplementary Fig. 24. Experimentally measured n_{min} values from the dwell time distributions at $T = 22^{\circ}C, 30^{\circ}C$, and $37^{\circ}C$.

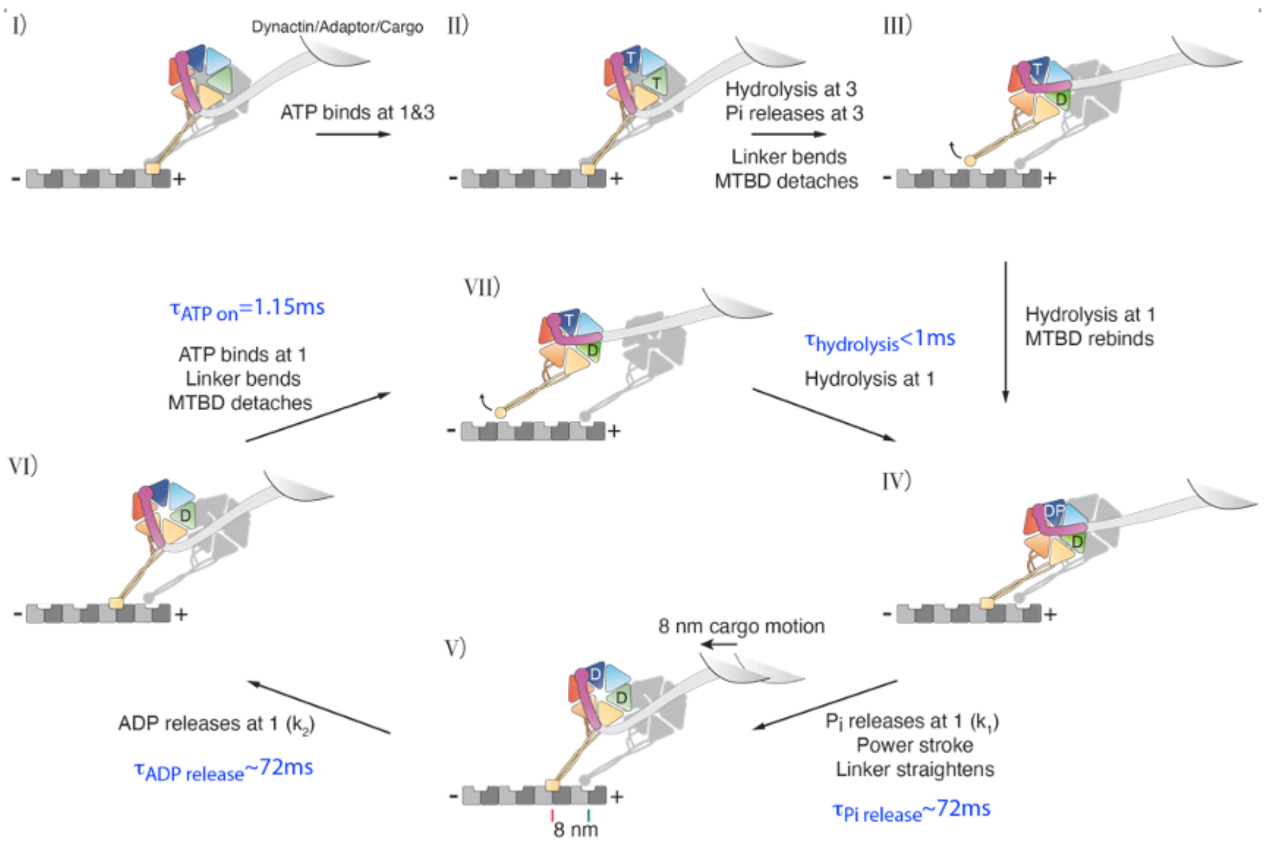

Supplementary Fig. 25. Schematic of the *previously proposed* active cycling-model where only ATP hydrolysis at AAA1 is required for dynein stepping. Most of the discussions on the active model focused on changes to the linker arm that were correlated with changes in the nucleotide states at the AAA binding sites. Here, we also show how these states may be linked to changes in the MTBD. **State (I)** has both AAA1 and AAA3 in the Apo state. The rectangular shape of the MTBD denotes the dynein motor domain strongly bound to the microtubule. The colored and grey dynein motor domain tails are attached to the dynactin/adaptor/cargo structure. **(I) → (II)**: ATP binds at AAA1 and leads to the rapid unbinding of dynein from the microtubule. **(II) → (III)**: With $ATP \rightarrow ADP \cdot Pi$ (denoted here as DP) on the AAA3 site, the dynein enters the weakly-bound state. In this state the linker arm contact to AAA5 is broken. **(III) → (IV)**: ATP hydrolyzes to $ADP \cdot Pi$ at AAA1, and the MTBD returns to the strongly bound state. **(IV→ V→VI → VII)**: The "active cycle" in which the ADP at AAA3 remain bound and the AAA1 site goes through cycles of $ADP \cdot Pi \rightarrow ADP \rightarrow Apo \rightarrow ATP$. If this model can explain our *in vivo* measurement, the Pi release at AAA1 is a possible assignment to our observed dwell time of 72 ms at $T = 22°C$. However, our measured dwell time is described by two equal rate constants, and the possible assignment of thermal desorption time $\tau_{ADP\ release} = 72\ ms$ of $ADP \rightarrow Apo$ is not compatible with kinetic measurements of ADP release in the presence of microtubules [15]. An additional difficulty is that the detachment force when ADP is on AAA3 suggests that the MTBD is in the weakly-bound state [4].

	Conditions	Mean backward force [pN]	95% confidence interval [pN]	MT affinity
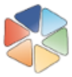	WT, apo	3.3	[3.1, 3.6]	S. B.
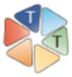	AAA1 E/Q + AAA3 E/Q, 1 mM ATP	0.9	[0.8, 0.9]	W. B.
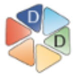	WT, 2 mM ADP	2.4	[2.3, 2.5]	W. B.
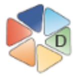	AAA1 K/A, 2 mM ADP	1.8	[1.6, 2.0]	W. B.
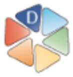	AAA3 K/A, 2 mM ADP	3.8	[3.5, 4.1]	S. B.

Supplementary Fig. 26. Single-molecule unbinding force of the dynein motor in different nucleotide states reported in Ref. [4]. S.B. and W.B. microtubule (MT) affinity stands for strongly-bound and weakly-bound states, respectively.

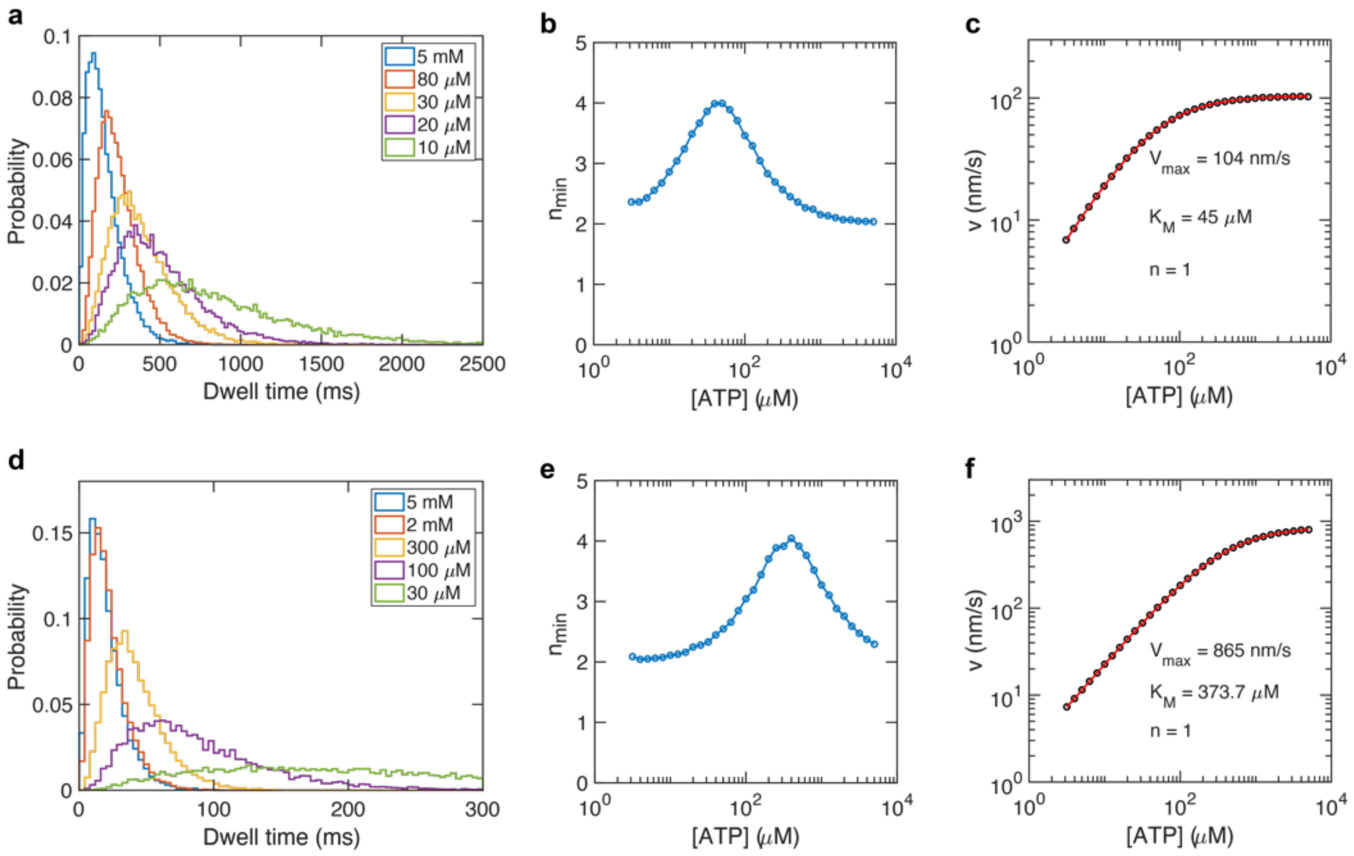

Supplementary Fig. 27. Block and Schnitzer have shown that the "randomness parameter" $r \equiv (\langle t^2 \rangle - \langle t \rangle^2)/\langle t \rangle^2$, the normalized distribution of dwell times in an enzymatic cycle is related to the number of kinetic sub-steps within a complete cycle[16]. For a sequence of M forward only steps with rate constants k_i, if p steps are comparable and the other $M - p$ rates are much higher, $r \sim 1/p$. In general $r^{-1} = n_{min} < M$, where n_{min} is the minimum number of kinetic steps of the full kinetic cycle[17].

In our live cell measurements, the two thermal desorption rates $ADP \cdot Pi \rightarrow ADP$ are the primary contributors to $r = 1/p \sim 1/2$, and all of the other rates are too fast to add to the dwell time dispersion. In the low $[ATP]$ regime, if the rate limiting step is due to ATP binding at *only* the AAA1[6,18], the *in vitro* experiments should give $r \sim 1$. If a dynein cycle requires 2 $ATPs$, $r \sim 1/2$. If the ATP concentration is adjusted so that the binding rates of ATP were comparable to the desorption rates of Pi's, the ATP on-rate the thermal desorption rates of Pi, $r \rightarrow 1/4$ if all of these rates are comparable. (Note that the ATP hydrolysis and the ADP desorption rates will remain too fast to contribute to r.)

A measurement of r as a function of $[ATP]$ would clearly differentiate between the 1-ATP active cycling model and our proposed 2-ATP model. The simulated dwell time distributions (a, d), randomness factors (b, e), and velocities (c, f) at various ATP concentrations, assuming the ATP binding rates and the Pi desorption rates at AAA1 and AAA3 are the same. The simulations were performed for $T = 22°C$ (a-c) and $T = 37°C$ (d-f). In the limit of low or high $[ATP]$ ($< 5\ \mu M$ or $> 1\ mM$) $n_{min} \rightarrow 2$. In order to clearly see the fast rise in the dwell-time distribution, sufficient time resolution and fine time binning of the histogram are required. As discussed in Supplementary Fig. 21, the parameters of a dwell-time distribution function can be determined independent of the choice of the histogram bin size by fitting the raw dwell times using Maximum Likelihood Estimates (MLE).

Supplementary Tables

Supplementary Table 1.

Preparation of silica-coated UCNPs.

Reagents	UCNP-1	UCNP-2	UCNP-3	UCNP-4
Igepal CO-520 (mg)	1000	500	1500	1500
UCNPs (μL)	200	100	333	100
Ammonia solution (μL)	150	150	70	70
TEOS (μL)	12	12	100	5

UCNP-1: 22 nm $NaYF_4$: 20% Yb, 2 % Er
UCNP-2: 29.4 nm $NaYF_4$@$NaYbF_4$: 8 % Er@$NaYF_4$
UCNP-3: 66.4 nm $NaYbF_4$: 8 % Er, 10% Gd@$NaYbF_4$: 8 % Er@$NaYF_4$
UCNP-4: 160 nm $NaYbF_4$: 10% Gd, 8% Er@$NaYbF_4$: 8% Er@$NaYbF_4$: 8% Er@$NaYF_4$

Supplementary References

1 Derr, N. D. *et al.* Tug-of-war in motor protein ensembles revealed with a programmable DNA origami scaffold. *Science* **338**, 662-665 (2012).

2 Elshenawy, M. M. *et al.* Cargo adaptors regulate stepping and force generation of mammalian dynein-dynactin. *Nature chemical biology* **15**, 1093-1101 (2019).

3 Leon-Garcia, A. in *Probability, Statistics, and Random Processes For Electrical Engineering* Ch. 7, 360 (Pearson, 2007).

4 Nicholas, M. P. *et al.* Cytoplasmic dynein regulates its attachment to microtubules via nucleotide state-switched mechanosensing at multiple AAA domains. *PNAS* **112**, 6371-6376 (2015).

5 DeWitt, M. A., Cypranowska, C. A., Cleary, F. B., Belyy, V. & Yildiz, A. The AAA3 domain of cytoplasmic dynein acts as a switch to facilitate microtubule release. *Nature structural & molecular biology* **22**, 73-80 (2015).

6 Reck-Peterson, S. L. *et al.* Single-molecule analysis of dynein processivity and stepping behavior. *Cell* **126**, 335-348 (2006).

7 Carter, A. P. *et al.* Structure and Functional Role of Dynein's Microtubule-Binding Domain. *Science* **322**, 1691-1695 (2008).

8 Nan, X., Sims, P. A. & Xie, X. S. Organelle tracking in a living cell with microsecond time resolution and nanometer spatial precision. *Chemphyschem : a European journal of chemical physics and physical chemistry* **9**, 707-712 (2008).

9 Peng, C. S. *et al.* Nanometer-Resolution Long-term Tracking of Single Cargos Reveals Dynein Motor Mechanisms. *bioRxiv*, 2022.2001.2005.475120 (2022).

10 Moffitt, J. R. *et al.* Intersubunit coordination in a homomeric ring ATPase. *Nature* **457**, 446-450 (2009).

11 Ross, J. L., Wallace, K., Shuman, H., Goldman, Y. E. & Holzbaur, E. L. F. Processive bidirectional motion of dynein–dynactin complexes in vitro. *Nature Cell Biology* **8**, 562-570 (2006).

12 Liu, Q. *et al.* Single upconversion nanoparticle imaging at sub-10 W cm2 irradiance. *Nature Photonics* **12**, 548-553 (2018).

13 Cui, B. *et al.* One at a time, live tracking of NGF axonal transport using quantum dots. *PNAS* **104**, 13666-13671 (2007).

14 Hayashi, K., Hasegawa, S., Sagawa, T., Tasaki, S. & Niwa, S. Non-invasive force measurement reveals the number of active kinesins on a synaptic vesicle precursor in axonal transport regulated by ARL-8. *Phys Chem Chem Phys* **20**, 3403-3410 (2018).

15 Holzbaur, E. L. F. & Johnson, K. A. Microtubules accelerate ADP release by dynein. **28**, 7010-7016 (1989).

16 Schnitzer, M. J. & Block, S. M. Statistical kinetics of processive enzymes. *Cold Spring Harb Symp Quant Biol* **60**, 793-802 (1995).

17 Moffitt, J. R. & Bustamante, C. Extracting signal from noise: kinetic mechanisms from a Michaelis-Menten-like expression for enzymatic fluctuations. *Febs j* **281**, 498-517 (2014).

18 DeWitt, M. A., Chang, A. Y., Combs, P. A. & Yildiz, A. Cytoplasmic dynein moves through uncoordinated stepping of the AAA+ ring domains. *Science* **335**, 221-225 (2012).
