# Supplementary material for: Nanometer-resolution tracking of single cargo reveals dynein motor mechanisms
Source: Nat Chem Biol. 2024 Aug 1;21(5):648–56. doi: 10.1038/s41589-024-01694-2 (PMC11785820; doi:10.1038/s41589-024-01694-2)
Supplement: Supplementary file 2 — Reporting Summary [file 41589_2024_1694_MOESM2_ESM.pdf]

nature portfolio

Corresponding author(s): Steven Chu

Last updated by author(s): 2024/05/12

Reporting Summary

Nature Portfolio wishes to improve the reproducibility of the work that we publish. This form provides structure for consistency and transparency in reporting. For further information on Nature Portfolio policies, see our Editorial Policies and the Editorial Policy Checklist.

Statistics

For all statistical analyses, confirm that the following items are present in the figure legend, table legend, main text, or Methods section.

n/a	Confirmed	
☐	☒	The exact sample size (*n*) for each experimental group/condition, given as a discrete number and unit of measurement
☐	☒	A statement on whether measurements were taken from distinct samples or whether the same sample was measured repeatedly
☒	☐	The statistical test(s) used AND whether they are one- or two-sided<br>*Only common tests should be described solely by name; describe more complex techniques in the Methods section.*
☒	☐	A description of all covariates tested
☐	☒	A description of any assumptions or corrections, such as tests of normality and adjustment for multiple comparisons
☐	☒	A full description of the statistical parameters including central tendency (e.g. means) or other basic estimates (e.g. regression coefficient) AND variation (e.g. standard deviation) or associated estimates of uncertainty (e.g. confidence intervals)
☒	☐	For null hypothesis testing, the test statistic (e.g. *F*, *t*, *r*) with confidence intervals, effect sizes, degrees of freedom and *P* value noted<br>*Give P values as exact values whenever suitable.*
☐	☒	For Bayesian analysis, information on the choice of priors and Markov chain Monte Carlo settings
☒	☐	For hierarchical and complex designs, identification of the appropriate level for tests and full reporting of outcomes
☒	☐	Estimates of effect sizes (e.g. Cohen's *d*, Pearson's *r*), indicating how they were calculated

Our web collection on statistics for biologists contains articles on many of the points above.

Software and code

Policy information about availability of computer code

Data collection	Andor Solis was used for data collection.
Data analysis	Matlab 2017b was used for data analysis. Fiji 2.14.0 was used to plot TEM images. The HMM-Bayes software written in MATLAB was downloaded from http://hmm-bayes.org/. Step sizes were determined by a step-finding program developed by Kerssemakers et al (ref. 80.)

For manuscripts utilizing custom algorithms or software that are central to the research but not yet described in published literature, software must be made available to editors and reviewers. We strongly encourage code deposition in a community repository (e.g. GitHub). See the Nature Portfolio guidelines for submitting code & software for further information.

Data

Policy information about availability of data

All manuscripts must include a data availability statement. This statement should provide the following information, where applicable:

- Accession codes, unique identifiers, or web links for publicly available datasets
- A description of any restrictions on data availability
- For clinical datasets or third party data, please ensure that the statement adheres to our policy

The main data supporting the findings of this study are included in the main article and its Supplementary Figures. Additional details on datasets and protocols will be available by the corresponding authors upon request.

Human research participants

Policy information about studies involving human research participants and Sex and Gender in Research.

Reporting on sex and gender	n/a
Population characteristics	n/a
Recruitment	n/a
Ethics oversight	n/a

Note that full information on the approval of the study protocol must also be provided in the manuscript.

Field-specific reporting

Please select the one below that is the best fit for your research. If you are not sure, read the appropriate sections before making your selection.

☒ Life sciences ☐ Behavioural & social sciences ☐ Ecological, evolutionary & environmental sciences

For a reference copy of the document with all sections, see nature.com/documents/nr-reporting-summary-flat.pdf

Life sciences study design

All studies must disclose on these points even when the disclosure is negative.

Sample size	For TEM experiments, more than 10 field of views were imaged to get hundreds of nanoparticles to determine the size distribution. Nanoparticle synthesis and functionalization was performed more than 3 times to ensure the robustness of the protocols. For in vitro experiments such as binding assays, more than 20 random field of views were imaged to determine the brightness of single UCNPs. More than 3 independent experiments were also performed. For neuron tracking experiments, each endosome was tracked over multiple field of views across a distance of several hundreds of micrometers. 12 different cargoes from 12 different neurons from three independent experiments were imaged to determine the phi distributions. For step-resolved experiments, one cargo was tracked over hundreds of molecule steps. Three different cargoes from different neuron cultures were tracked for the temperature-dependent study.
Data exclusions	In ~ 60% of the approximately constant velocity movements, ϕ approached a steady-state value and we focus our analysis on this subset of trajectories. The other ~40% trajectory fragments were excluded.
Replication	Experiments were performed over several months and replicated in more than 3 independent experiments. Details for different types of experiments are stated in the "Sample size" section.
Randomization	All chemical reagents were aliquoted into small volumes and randomly used in different experiments. Random fields of views were imaged.
Blinding	Imaging experiments were inherently blinded. Random cargoes were tracked over hundreds of micrometers. Data analysis was performed later to extract the phi distribution. For step-resolved experiments, cargoes moving at lower speed was tracked to better resolve the steps. Nanoparticle synthesis and functionalization experiments are also inherently blinded. The phase and ion compositions of nanoparticles were measured using XRD and ICP after the synthesis to confirm the identity of the nanoparticles.

Reporting for specific materials, systems and methods

We require information from authors about some types of materials, experimental systems and methods used in many studies. Here, indicate whether each material, system or method listed is relevant to your study. If you are not sure if a list item applies to your research, read the appropriate section before selecting a response.

Materials & experimental systems

n/a	Involved in the study	
☒	☐	Antibodies
☐	☒	Eukaryotic cell lines
☒	☐	Palaeontology and archaeology
☐	☒	Animals and other organisms
☒	☐	Clinical data
☒	☐	Dual use research of concern

Methods

n/a	Involved in the study	
☒	☐	ChIP-seq
☒	☐	Flow cytometry
☒	☐	MRI-based neuroimaging

Eukaryotic cell lines

Policy information about cell lines and Sex and Gender in Research

Cell line source(s)	HeLa (ATCC CCL-2). Primary embryonic rat dorsal root ganglion cells. Human induced neurons from human embryonic stem cells (WA01/H1 cell line, NIH registry 0043).<br>H1 human embryonic stem (ES) cells (WA01; RRID: CVCL_9771; NIH Approval Number: NIHhESC-10-0043) were obtained from WiCell Research Resources (Wicell, WI), maintained in feeder-free condition using mTeSR1 medium (Stem Cell Technologies), and used at intermediate (~50) passage numbers to generate human induced neuronal (iN) cells (Zhang et al., 2013). They are routinely analyzed for 1) characteristic hiPSC cell morphology, 2) expression of pluripotency markers OCT4, NANOG, and SOX2, 3) ability to differentiate in vitro into multiple cell types, and 4) a normal complement of 46 chromosomes by karyotyping (every 20 passages). These standards are consistent with established guidelines for maintenance of hPSCs (Stem Cells. 2006 Jan;24(1):145-50.; Stem Cells Transl Med. 2015 Mar;4(3):217-23.)
Authentication	Cell line authentication was not performed. All cell lines were expanded from the original vials vendors provided.
Mycoplasma contamination	Mycoplasma contamination was not tested.
Commonly misidentified lines<br>(See ICLAC register)	We did not use commonly misidentified lines listed by ICLAC.

Animals and other research organisms

Policy information about studies involving animals; ARRIVE guidelines recommended for reporting animal research, and Sex and Gender in Research

Laboratory animals	Dorsal Root Ganglion (DRG) neurons were harvested from Sprague-Dawley rats (Charles River Laboratories). The Sprague-Dawley rats were ordered as timed pregnant rats. The age of the mother rats was not specified but in the range of 4-8 months. Each dissection used one mother rat, which had 8 embryos on average. The results reported in this paper came from about 10 dissections at embryonic day 18 (E18).
Wild animals	This study did not involve wild animals.
Reporting on sex	n/a; We did not keep track of the sex of the rat embryos. We are not aware if axonal transport depends on sex based on the literature.
Field-collected samples	This study did not involve field samples.
Ethics oversight	Dorsal Root Ganglion (DRG) neurons were harvested from Sprague-Dawley rats (Charles River Laboratories) using an experimental procedure approved by the animal ethics committee - Panel for Laboratory Animal Care of Stanford University (APLAC-20608), in accordance with Stanford University's IACUC policies for the use of animals in research.

Note that full information on the approval of the study protocol must also be provided in the manuscript.
